# Late domain dependent E-cadherin recruitment into extracellular vesicles

**DOI:** 10.3389/fcell.2022.878620

**Published:** 2022-09-07

**Authors:** Sebastian Bänfer, Sophie Kutscher, Fenja Fleck, Martina Dienst, Christian Preußer, Elke Pogge von Strandmann, Ralf Jacob

**Affiliations:** ^1^ Department of Cell Biology and Cell Pathology, Philipps University Marburg, Marburg, Germany; ^2^ Center for Tumor Biology and Immunology (ZTI), Institute for Tumor Immunology, Philipps University Marburg, Marburg, Germany

**Keywords:** E-cadherin, exosomes, late domain, ESCRT (endosomal sorting complex required for transport), multivesicular bodies (MVB), extracellular vesicles

## Abstract

E-cadherin, a transmembrane protein involved in epithelial cell-cell adhesion and signaling, is found in exosomal fractions isolated from human body fluids. A cellular mechanism for recruitment of E-cadherin into extracellular vesicles (EVs) has not yet been defined. Here, we show that E-cadherin is incorporated into the membrane of EVs with the extracellular domain exposed at the vesicle surface. This recruitment depends on the endosomal sorting complex required for transport I (ESCRT-I) component Tsg101 and a highly conserved tetrapeptide P(S/T)AP late domain motif in the cytoplasmic tail of E-cadherin that mediates interaction with Tsg101. Mutation of this motif results in a loss of interaction and a dramatic decrease in exosomal E-cadherin secretion. We conclude, that the process of late domain mediated exosomal recruitment is exerted by this endogenous non-ESCRT transmembrane protein.

## Introduction

EVs are extracellular vesicles with diameters ranging from 30–150 nm (van Niel et al., 2018). They are generated within the endosomal system as intraluminal vesicles (ILVs) and secreted during the fusion of multivesicular endosomes (MVEs) with the plasma membrane into the outer milieu. Almost every cell type can secrete EVs under physiological or pathological conditions. Their cargo composition is manifold and cell-type specific. Accordingly, cellular machinery to recruit cargo into ILVs is diverse. A prominent example is the endosomal sorting complex required for transport (ESCRT), which acts stepwise in the formation of MVEs and ILVs. Alternative ILV-sorting mechanisms employ the generation of ceramide by neutral type II sphingomyelinase, which hydrolyses sphingomyelin to ceramide (Trajkovic et al., 2008). The members CD9, CD63, CD81 and CD82 of the tetraspanin family have also been shown to be involved in ESCRT-independent cargo-sorting to EVs by the formation of dynamic membrane microdomains (Theos et al., 2006; van Niel et al., 2011; Charrin et al., 2014; Gauthier et al., 2017). Moreover, clustering of the cytosolic adaptor syntenin and the auxiliary component ALIX with the transmembrane proteoglycan syndecan supports their EV recruitment (Baietti et al., 2012). The observation that syntenin also controls Arf6-mediated syndecan-recycling through endosomal compartments emphasizes interconnectivity of vesicular pathways for endocytic recycling and exosomal recruitment. This is further evidenced by the aggregation-dependent rerouting of the transferrin receptor from membrane-recycling to sorting into EVs (Vidal et al., 1997).

E-cadherin is a membrane-anchored glycoprotein that couples calcium-dependent cell-cell adhesion to the cytoskeleton and intracellular signaling pathways in epithelial cells. Upon destabilization of intercellular adhesion by depletion of extracellular Ca^2+^ ions E-cadherin is endocytosed into endosomal vesicles (Kartenbeck et al., 1991). Some of these actively internalized E-cadherin polypeptides are then recycled back to the basolateral plasma membrane (Le et al., 1999). Lock and Stow showed that newly synthesized as well as endocytosed E-cadherin traverses Rab11-positive recycling endosomes before entering the plasma membrane (Lock and Stow, 2005). Their observations indicate a constant uptake of small quantities of E-cadherin in epithelial monolayers with a markedly increase in E-cadherin-endocytosis following destabilization of cell-cell contacts. The endocytic uptake of E-cadherin itself depends on the formation of clathrin coats and is regulated by AP2 and clathrin recruitment as well as the concerted action of the formin Diaphanous and Myosin-II (Levayer et al., 2011). Although the main cellular functions of E-cadherin are exerted at the plasma membrane of epithelial cells, there is accumulating evidence for extracellular E-cadherin in human body fluids. E-cadherin can be shed from the plasma membrane by proteolytic cleavage as soluble E-cadherin (Grabowska and Day, 2012) or recruited to the exosomal membrane (Zhang et al., 2020). Tang et al. (2018) have recently shown that EVs exposing E-cadherin can induce angiogenesis *in vitro* and *in vivo* by a crosstalk between the nuclear factor-κB (NFκB) and β-catenin signaling cascades. The newly formed vasculature then leads to ovarian cancer progression and metastasis. However, cellular components involved in E-cadherin recruitment into EVs have remained elusive.

We now report data showing that in epithelial MDCK cells a small proportion of E-cadherin is recruited into ILVs, which are secreted at the plasma membrane as EVs. Recruitment involves interaction of a highly conserved P(S/T)AP late domain motif in the cytoplasmic tail of E-cadherin with the ESCRT I component Tsg101. Mutagenesis of this motif or Tsg101-knockdown reduce the recruitment efficiency of E-cadherin into EVs. In addition to the previously published observation of soluble cargo recruitment (Bänfer et al., 2018), this study suggests that P(S/T)AP late domains are also involved in the recruitment of endogenous membrane-anchored polypeptides into EVs.

## Materials and methods

### DNA constructs

Canine E-cadherin inserted into the peGFP-N1 vector was kindly provided by W. James Nelson (Adams et al., 1998). Cadherin domains 2 to 5 (amino acids 272–671) were deleted by two-step mutagenesis PCR using the primer pair 5′- CAC​CCA​GGC​AGT​CTT​CCA​AGG​ATA​TCT​CAA​GCT​CAC​AGA​TAA​CC -3′ and 5′- GGT​TAT​CTG​TGA​GCT​TGA​GAT​ATC​CTT​GGA​AGA​CTG​CCT​GGG​TG -3′ to generate plasmid pE-cadherinΔE2-5GFP_PTAP_. Mutation of the E-cadherin PTAP motif into ASAA was induced by overlap extension PCR with inside primers 5′- CG GAC ACT GAC gCT Agc GCT gCT CCT TAT GAC-3′ and 5′- GTC ATA AGG AGc AGC gcT AGc GTC AGT GTC CG-3’ (mutated nucleotides are depicted in small letters). Successful generation of plasmid constructs was validated by sequence analysis.

### Antibodies and nanobodies

The following monoclonal and polyclonal antibodies or nanobodies were used in this study: anti-E-cadherin (C-terminus: BD Transduction Laboratories, 61018; N-terminus: Genetex/Biozol GTX134997), anti-Tsg101 (Abcam, 4A10), anti-GFP (Takara, 632592), anti-GFP-nanobodies (Chromotek), anti-actin (BD Transduction Laboratories, 612656), anti-Hrs (Enzo, A-5; GeneTex, GTX89364), anti-GAPDH (Abcam, 6C5), anti-giantin (Covance, PRB-114C), anti-PDI (BD Transduction Laboratories, 610946), anti-TOM20 (Santa Cruz, Sc11415).

### Alignment and sequence logos

E-cadherin sequences were aligned with ClustalOmega (McWilliam et al., 2013). Sequence logos aligned to the decapeptide (765)DTPTAPPYD(773) were generated by WebLogo (Crooks et al., 2004).

### Cell culture and transfection

MDCK (Madin-Darby Canine Kidney) type II cells were cultured in MEM high glucose supplemented with 2 mM glutamine, 100 U/ml penicillin, 100 mg/ml streptomycin and 10% FCS at 37°C in humidified atmosphere containing 5% CO_2_. Transfections were performed with Lipofectamine 2000 (Invitrogen). For generation of stable cell lines, MDCK cells were split in high ratios 2 days after transfection and selected in MEM medium containing 0.4 mg/ml Zeocin or an equivalent antibiotic for selection. We transferred single clones to 24 well plates with Trypsin/EDTA-soaked Whatman slices. Subsequently, the clones were analyzed for expression of the exogenous proteins by immunoblot and fluorescence microscopy. Only those clones were selected that exhibited a transfection efficiency of at least 90% transfected cells.

### RNA interference

Cells were transfected as previously described (Bänfer et al., 2018) at day 1 after seeding with the following siRNA duplexes (Invitrogen): Tsg101: 5′-GGU UAC CCG UUU AGA UCA A [dT][dT]-3′, 5′-UUG AUC UAA ACG GGU AAC C [dT][dT]-3’; Hrs: 5′-UUC UUC UCC CAG UAG UUC C [dT][dT]-3′, 5′-GGA ACU ACU GGG AGA AGA A [dT][dT]-3′ and 5′-GGA ACG AGC CCA AGU ACA A [dT][dT]-3′, 5′-UUG UAC UUG GGC UCG UUC C [dT][dT]-3’.

### Preparation of extracellular vesicless

The cells were washed three times with PBS and incubated overnight with MEM and 10% fetal calf serum (FCS). To avoid contamination of the exosomal fraction by bovine serum EVs, cell culture media were subjected to centrifugation at 100,000 g for 2 h prior to overnight incubation. Medium was collected and submitted to a series of centrifugation steps as previously described (Bänfer et al., 2018). Cell culture supernatants were centrifuged at 100,000 g for 1 h. The resulting pellet was washed in PBS++ (PBS supplemented with 1 mM CaCl2 and 1 mM MgCl2), repelleted again at 100,000 g for 1 h and then resuspended in either PBS++ or in SDS-PAGE sample buffer for further use. All steps were performed at 4°C.

### Proteinase K protection assay

Exosomal pellets were resuspended in PBS++, pooled, and subsequently split into three identical aliquots. Proteinase digestion was then performed with 0.5 mg/ml to 1 mg/ml proteinase K (Fermentas) in presence or absence of 1% Triton X-100 for 30 min at 37°C. As control, one of the aliquots was incubated without proteinase K.

### Co-immunoprecipitation

MDCK cells stably expressing eGFP or eGFP fusion proteins were washed with PBS^++^, followed by mechanical detachment of the cells in PBS^++^. The cells were pelleted by centrifugation at 500 *g* for 3 min at 4°C and rinsed twice with PBS^++^. Cell lysis was achieved by application of lysis buffer (10 mM HEPES, 150 mM NaCl, 0.5 mM EDTA, 1% Triton X-100, 0.5% SDS and proteinase inhibitor cocktail, pH 7.5). Cell lysates (17,000 *g*, 15 min, 4°C) were then incubated with GFP-nanobody agarose (GFP-Trap, Chromotek) or control beads without GFP-nanobodies for 1.5 h at 4°C. Finally, beads were rinsed four times with Co-IP washing buffer (10 mM HEPES, 150 mM NaCl, 0.5 mM EDTA) and boiled in SDS-PAGE loading buffer for Western blot analysis.

### Immunostaining, immunofluorescence microscopy and image processing

Immunofluorescence analysis was performed essentially as previously described (Bänfer et al., 2018). The cells were grown on cover slips and fixed with 4% paraformaldehyde for 20 min. Afterwards, cells were permeabilized with 0.1% Triton-X-100 for 20 min and blocked in 5% BSA/PBS^++^ for 1 h. Primary antibodies were added in blocking reagent for 2 h or overnight. Secondary antibodies labelled with the indicated Alexa Fluor dyes were applied in PBS^++^ for 1 h. Nuclei were stained with Hoechst 33342. Following incubation, cells were washed with PBS^++^ and mounted with Mowiol. Confocal images were acquired on a Leica STELLARIS microscope equipped with a ×93 glycerol planapochromat objective (Leica Microsystems). Processing of images was done with Leica LAS X and Volocity 5 (PerkinElmer). We calculated co-localization between markers as Manders’ coefficient using the Volocity software package. Structures with coefficients <0.5 were classified as “not-colocalized”.

### Nano-flow cytometry

For nanoFCM, a Nano Analyzer (NanoFCM Co. Ltd., Nottingham, United Kingdom) equipped with a 488 nm laser, was calibrated using 200 nm polystyrene beads (NanoFCM Co.) with a defined concentration of 2.08 × 108 particles/ml, which were also used as a reference for particle concentration. In addition, monodisperse silica beads (NanoFCM Co. Ltd.) of four different sizes served as size reference standards to calibrate the size of EVs. Freshly filtered (0.1 µm) 1 × PBS was analyzed as background signal and subtracted from the other measurements. Each dot plot was derived from data collected approximately 4,000 events with a sample pressure of 1.0 kPa. For immunofluorescence staining, the following antibodies were used (BioLegend): FITC-conjugated mouse anti-human/canine CD9 antibody (clone HI9a), and anti-human Ecad, with secondary PE-conjugated donkey anti-rabbit IgG antibody (clone Poly4064); as isotype controls, FITC-conjugated mouse IgG1, κ (clone MOCP-21), and PE-conjugated donkey anti-rabbit IgG antibody (clone Poly4064); 1 ng/µl of each antibody in 50 µL 1 × PBS. After removing antibody aggregates by centrifugation at 12,000× g for 10 min, the supernatant was added to 5 × 108 purified EVs, followed by incubation for 90 min at 25°C under constant shaking. Stained EV were diluted 1:100 in 1xPBS for NanoFCM analysis.

## Results

### E-cadherin is recruited into extracellular vesicless and interacts with Tsg101

**FIGURE 1:**
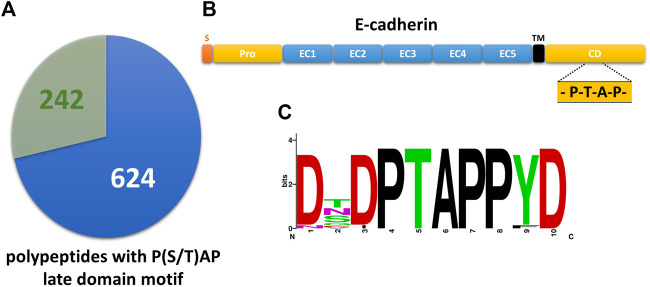
The cytoplasmic tail of E-cadherin comprises a highly conserved PTAP-late domain motif. **(A)** Pie chart showing 242 primary polypeptide sequences of the UniProtKB/Swiss-Prot database that contain a P(S/T)AP late domain motif and are listed in ExoCarta (see also Supplementary Table). 624 late domain containing sequences from the UniProtKB/Swiss-Prot database are not listed in ExoCarta. **(B)** Domain structure of E-cadherin. S, signaling sequence; pro, pro-domain; EC, E-cadherin domain; TM, transmembrane domain; CD, cytoplasmic domain. The location of the PTAP late domain motif is indicated. **(C)** Alignment of E-cadherin late domain-like motifs found in 32 vertebrates was used to generate a sequence logo of the decapeptide (765)DTPTAPPYD(773).

**FIGURE 2:**
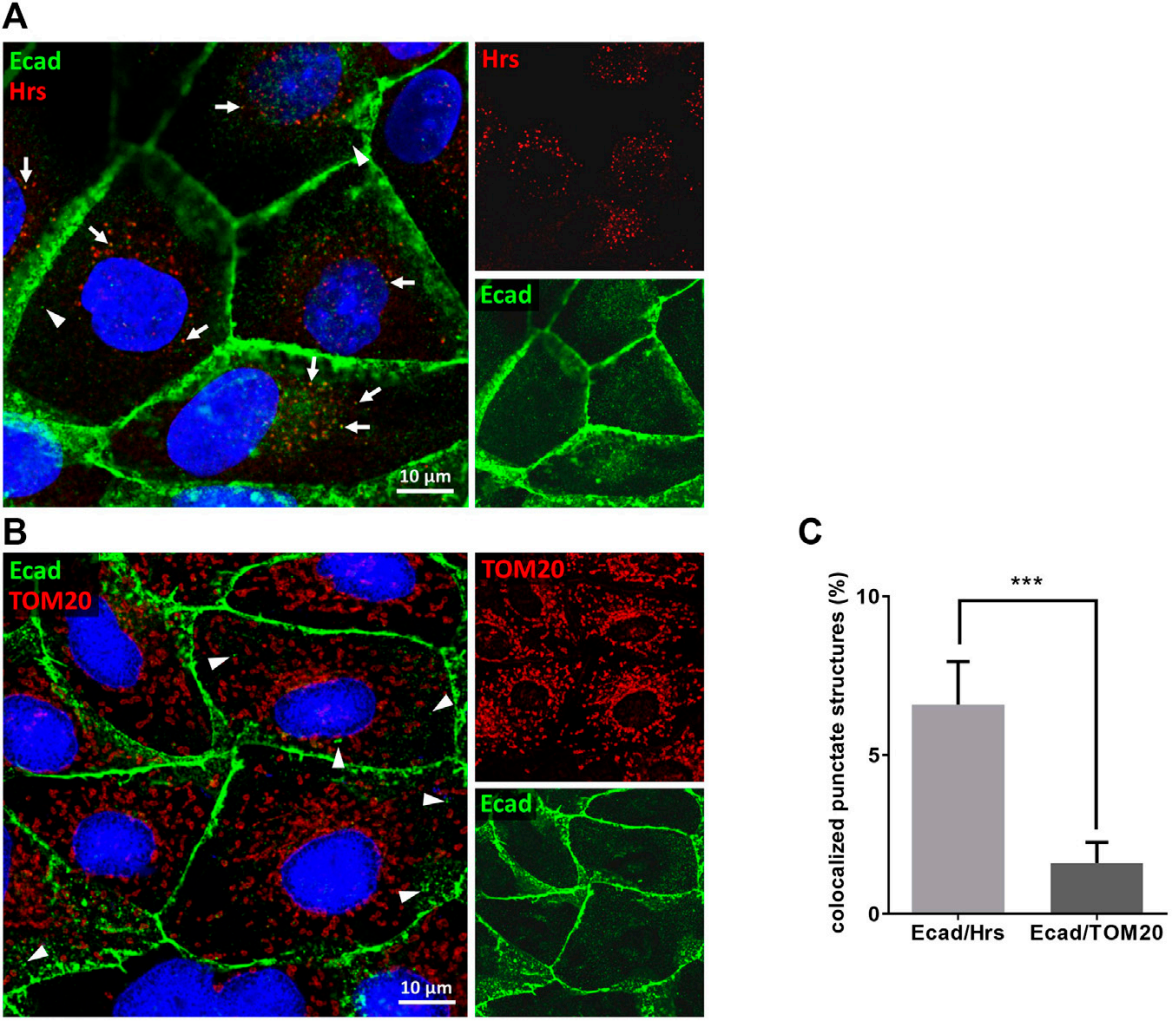
E-cadherin colocalizes with Hrs in MDCK cells. Confocal codistribution analysis of immunostained E-cadherin with the MVE-protein Hrs **(A)** or mitochondrial outer membrane protein TOM20 **(B)**. MDCK cells were cultivated for 2 days, fixed and stained by immunofluorescence with mAb anti-E-cadherin/Alexa Fluor 555 and pAb anti-Hrs/Alexa Fluor 647 or pAb anti-TOM20/Alexa Fluor 647. Nuclei are depicted in blue. Co-stained vesicular structures are indicated by arrows. Arrowheads point at punctate E-cadherin-positive structures that are not co-stained with Hrs or TOM20. **(C)** Manders’ correlation coefficient was used for quantification of experiments. Means ± s.e.m., 15–20 cells per experiment, *n* = 3 independent experiments. Nuclei were excluded from quantification. Statistical analysis in this figure: Student’s unpaired *t*-test, ***p < 0.001.

A PROSITE database search of the UniProtKB/Swiss-Prot database revealed 866 candidate polypeptides that contain at least one P(S/T)AP late domain motif (Figure 1A, Supplementary Table S1). 242 of these protein candidates are listed in the ExoCarta database as EV cargo proteins including 16 transmembrane proteins. Among them 5 candidates belong to the cadherin family. Primary sequences of E-cadherin orthologs revealed a conserved PS/TAP motif in the cytosolic domains of e.g., human (… P_767_T_768_A_769_P_770_ … ) and dog (… P_825_T_826_A_827_P_828_ … ) E-cadherin (Figures 1B,C). To assess if E-cadherin is recruited into ILVs of MVEs that can be released as EVs, epithelial MDCK cells were immunostained for the ESCRT-0 protein Hrs, which labels MVEs (Bache et al., 2003). About 6% of these Hrs-positive structures were co-stained with endogenously expressed E-cadherin, which is significantly above the co-staining efficiency of E-cadherin with mitochondrial TOM20 as negative control (Figure 2). These observations document that a small fraction of E-cadherin is closely related to MVEs, which are the source compartment for exosomal release.

**FIGURE 3:**
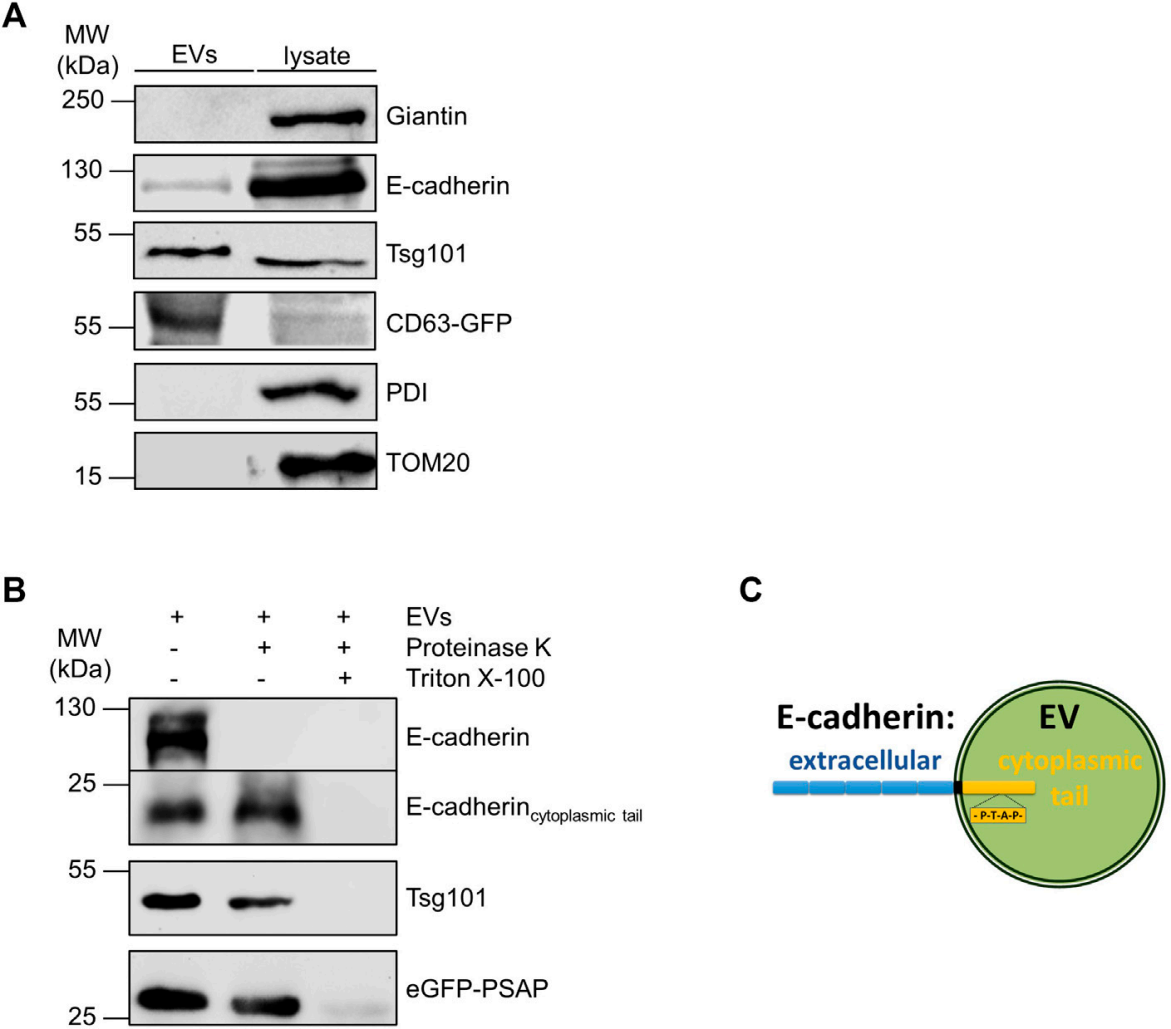
Identification of E-cadherin on isolated EVs. **(A)** Western blotting analysis of EV fractions and cell lysates. Representative results, *n* = 3 independent experiments. EVs and the corresponding cell lysates were analyzed by immunoblot with antibodies directed against E-cadherin and Tsg101. Antibodies directed against giantin (Golgi), PDI (ER) and TOM20 (mitochondria) were used as negative controls to validate the purity of EV-isolation. EVs isolated from MDCK_CD63-GFP_ cells were immunoblotted with anti-GFP antibodies and used as positive controls. **(B)** Proteinase protection assay. Antibodies directed against the cytoplasmic tail of E-cadherin were used for E-cadherin staining. Antibodies directed against Tsg101 and GFP were used as indicated. Representative results, *n* = 3 independent experiments. **(C)** Schematic drawing of the orientation of E-cadherin in the exosomal membrane. Schematic drawing of the orientation of E-cadherin in the membrane of an extracellular vesicle (EV). The extracellular part of the protein is shown in blue, and the part directed to the interior of the vesicle is shown in yellow.

We then isolated EVs from MDCK cell medium by differential centrifugation as previously published (Bänfer et al., 2018) and monitored the presence of E-cadherin in the EV fraction by immunoblot. Antibodies directed against Tsg101 were used as positive control for the validation of successful EV isolation. Figure 3A indicates that E-cadherin as well as Tsg101 are enriched in isolated EVs. Antibodies directed against endoplasmic reticulum protein disulfide isomerase (PDI), the Golgi component giantin and mitochondrial TOM20 were used as negative controls to verify the purity of isolated eEVs. To clarify the orientation of E-cadherin with its single transmembrane domain on the EV membrane, purified EVs were treated with the unspecific protease proteinase K in the presence or absence of Triton X-100 (Figure 3B). Antibodies directed against E-cadherin did not detect full length E-cadherin after proteinase K-treatment. This indicates, that the N-terminal extracellular domain of E-cadherin was accessible for the protease and therefore degraded. Others have shown that a significant fraction of E-cadherin can be proteolytically shed into the extracellular space through cleavage by secretases and caspases into 80 kDa soluble E-cadherin (sE-cadherin) and the remaining membrane-attached cytoplasmic tail of about 22 kDa (David and Rajasekaran, 2012). We also detected this smaller band with an antibody directed against the cytoplasmic domain of E-cadherin and this band was insensitive to proteinase K digestion. It was solely degraded if EV-membrane lipids were solubilized by Triton X-100. It is important to note that Tsg101 and the designed eGFP variant containing the late domain motif at the C-terminus (eGFP-PSAP), which reside in the lumen of EVs (Bänfer et al., 2018), showed a similar sensitivity pattern in this assay. Thus, E-cadherin is oriented with the large extracellular domain facing the outer surface of EVs while the cytoplasmic tail points into the EV lumen (Figure 3C). In essence, the cytoplasmic tail of E-cadherin, which harbors a PS/TAP motif, soluble eGFP-PSAP and the ESCRT-component Tsg101 are found in the EV lumen.

**FIGURE 4:**
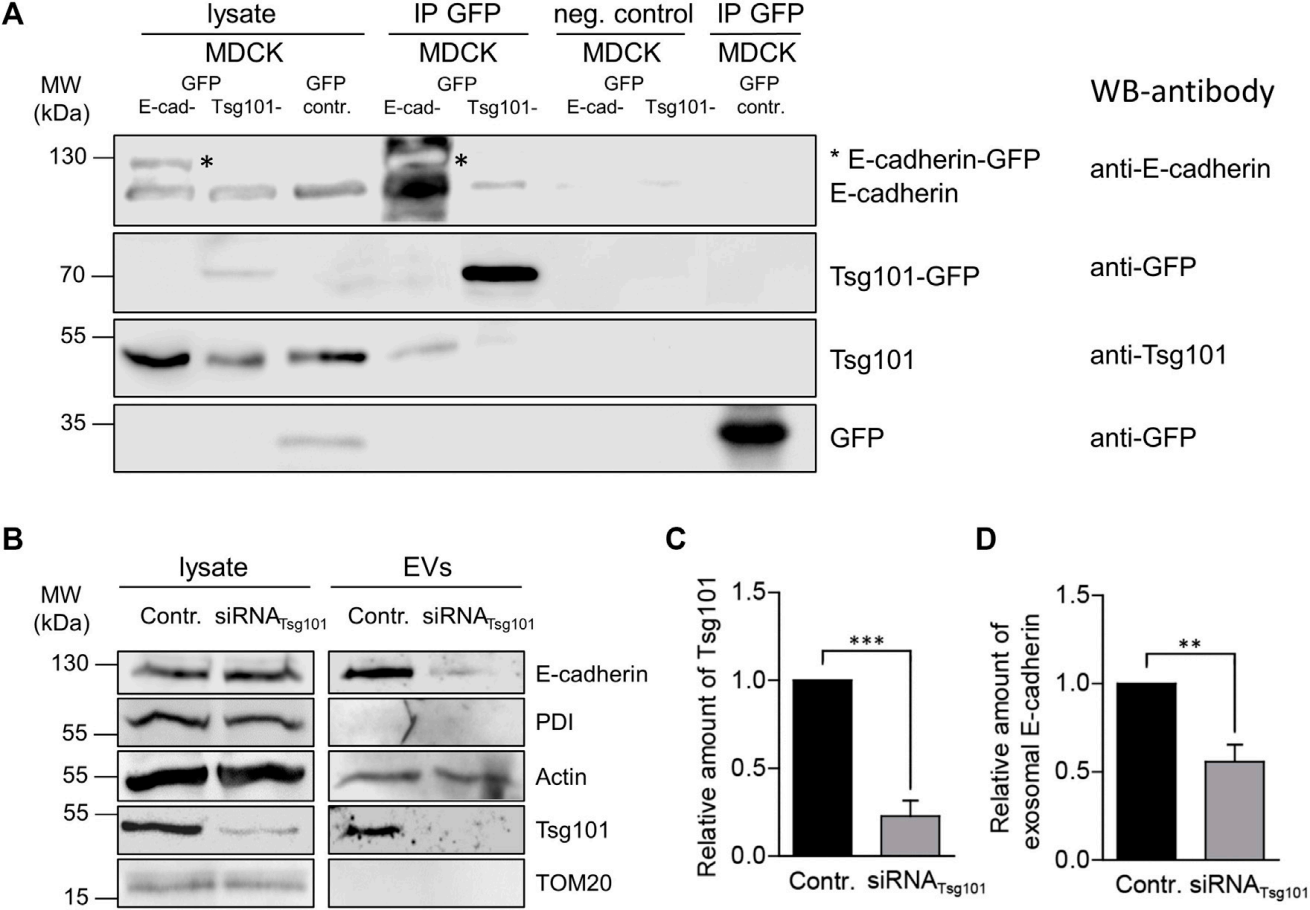
Tsg-101 dependent EV recruitment of E-cadherin. **(A)** Co-immunoprecipitation of E-cadherin-GFP or Tsg101-GFP from MDCK cell lysates. E-cadherin-GFP or Tsg101-GFP fusion proteins and their binding partners were immunoprecipitated with GFP-nanobody beads and detected by immunoblot using antibodies directed against E-cadherin, GFP and Tsg101. Non-specific precipitation was monitored by using control beads without nanobodies (neg. control) and GFP from MDCK_GFP_ cells (GFP-contr.). Antibodies used for western blots (WB) are indicated. Representative results, *n* = 3 independent experiments. **(B)** Tsg101 knockdown in MDCK cells and the corresponding EVs isolated from cell culture media. EVs and the corresponding cell lysates were analyzed by immunoblot using antibodies directed against E-cadherin and Tsg101. Antibodies directed against PDI were used as negative controls to validate the purity of EV-isolation. Actin was monitored as EV cargo molecule. **(C)** Efficient knockdown through Tsg101 siRNA administration was verified by quantification of Tsg101 in cell lysates from 3 independent experiments. Tsg101 quantities were normalized to GAPDH. Means ± s.e.m. **(D)** Quantification of the immunoblot analysis of the exosomal fraction after Tsg101 knockdown in MDCK cells as in **(C)**. E-cadherin was significantly reduced in Tsg101 siRNA-treated cells. Normalized to the E-cadherin quantities in the respective cell lysates. Means ± s.e.m., *n* = 3 independent experiments. Statistical analysis in this figure: Student’s unpaired *t*-test, ***p < 0.001; **p < 0.005; *p < 0.01.

Next, we sought to study putative interaction of E-cadherin with Tsg101. Therefore, we incubated MDCK_Ecad-GFP_ and MDCK_Tsg101-GFP_ cells stably expressing E-cadherin-GFP or Tsg101-GFP (Bänfer et al., 2018). GFP-Trap beads were used for precipitation of the GFP fusion proteins from cell lysates. Indeed, Tsg101 was pulled down by E-cadherin-GFP (Figure 4A). Moreover, Tsg101-GFP precipitated E-cadherin. This is a first hint for interaction between E-cadherin and Tsg101 in MDCK cells. However, we cannot conclude from these experiments, whether the interaction is direct or indirect.

In order to find out if Tsg101 plays a functional role in recruitment of E-cadherin into EVs, which could be in analogy to the mechanism published for soluble galectin-3 (Bänfer et al., 2018), we performed experiments were siRNA was used to specifically deplete the cellular content of Tsg101. Figures 4B,C show specific depletion to about 25% of residual Tsg101. Under these conditions EV recruitment of E-cadherin significantly declined by about 50% (Figures 4B,D). The EV-pool of actin, which has also been listed as EV cargo molecule (Xu et al., 2016), was not dramatically affected by Tsg101-depletion indicating that the cells still secrete representative EV quantities. These data suggest that expression of Tsg101 is linked to efficient incorporation of E-cadherin into secreted EVs, most likely by recognition of a cytoplasmic binding motif and the initiation of E-cadherin transport into budding ILVs.

### EV recruitment of E-cadherin with a mutated late domain motif

**FIGURE 5:**
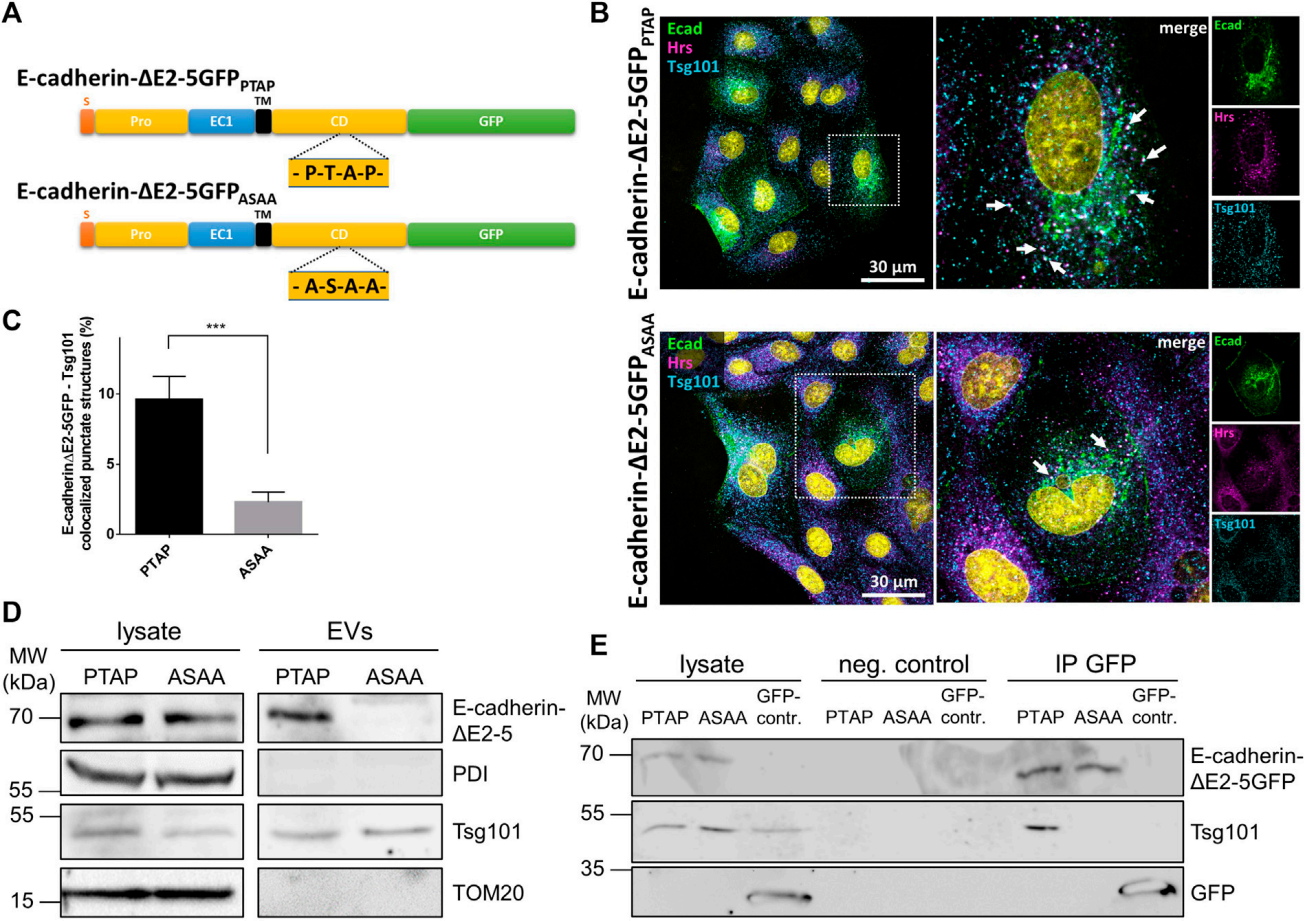
Subcellular localization and EV recruitment of E-cadherin variants. **(A)** Schematic drawing of the two E-cadherin deletion constructs. S, signaling sequence; pro, pro-domain; EC, E-cadherin domain; TM, transmembrane domain; CD, cytoplasmic domain. The location of the PTAP late domain motif and the mutagenized ASAA stretch are indicated. **(B)** Confocal codistribution analysis of MDCK cells transiently transfected with E-cadherinΔE2-5GFP or E-cadherinΔE2-5GFP_ASAA_ with the MVE-proteins Hrs and Tsg101. The cells were cultivated for 2 days post transfection, fixed and stained by immunofluorescence with mAb anti-Tsg101/Alexa Fluor 555 and pAb anti-Hrs/Alexa Fluor 647. Enlarged views of areas encircled by dotted lines are depicted on the right. Nuclei are depicted in cyan. Vesicular structures co-stained for E-cadherin, Hrs, and Tsg101 are indicated by arrows. **(C)** Colocalization of the two E-cadherinΔE2-5GFP variants and Tsg101 was estimated from at least 15 cells per experiment *via* the Manders’ colocalization coefficient. Data are represented as means ± s.e.m., significance was tested with Student’s *t*-test (***p < 0.001), *n* = 3 independent experiments. **(D)** EVs isolated from MDCK_E-cadherinΔE2-5GFPPTAP_ (PTAP) and MDCK_E-cadherinΔE2-5GFPASAA_ (ASAA) culture media and the corresponding cell lysates were analyzed by immunoblot using antibodies directed against E-cadherin and Tsg101. Antibodies directed against PDI and TOM20 were used as negative controls to validate the purity of EV-isolation. **(E)** Co-immunoprecipitation of E-cadherin variants with Tsg101 from MDCK_E-cadherinΔE2-5GFPPTAP_ (PTAP) and MDCK_E-cadherinΔE2-5GFPASAA_ (ASAA) cell lysates. E-cadherinΔE2-5GFP or E-cadherinΔE2-5GFP_ASAA_ and their binding partners were immunoprecipitated with GFP-nanobody beads and detected by immunoblot using antibodies directed against GFP or Tsg101. Non-specific precipitation was monitored by using control beads without nanobodies (neg. control) and GFP from MDCK_GFP_ cells (GFP-contr.).

We thus addressed the question if the cytoplasmic PTAP late domain motif of E-cadherin mediates ILV-recruitment of E-cadherin and interaction between E-cadherin and Tsg101. To increase the transfection efficiency and to facilitate the generation of stable cell clones, we first generated a GFP-tagged variant of E-cadherin with a deletion of cadherin domains two to five (E-cadherinΔE2-5GFP_PTAP_) (Figure 5A). In a second step, the cytoplasmic PTAP motif of this variant was mutated to ASAA (E-cadherinΔE2-5GFP_ASAA_), mimicking a mutation that is known to abrogate the release of Marburg virus-like particles (Dolnik et al., 2010). The two constructs were transiently transfected into MDCK cells. 48 h after transfection the cells were fixed and immunostained for Hrs and Tsg101 as MVE-markers (Figure 5B). Quantification using Manders’ correlation coefficients revealed that 9.61 ± 1.7% of cytoplasmic E-cadherinΔE2-5GFP_PTAP_ structures localized to Tsg101-positive vesicles representing the MVB formation site (Figures 5B,C). In contrast, co-staining of E-cadherinΔE2-5GFP_ASAA_ resulted in a significantly reduced overlap of 2.30 ± 0.7%, thus supporting the idea of a relevant role of the cytoplasmic PTAP late domain in ILV-sorting of this transmembrane polypeptide. Consequences of PTAP late domain mutation on EV recruitment of E-cadherin were studied in MDCK cells stably expressing E-cadherinΔE2-5GFP (MDCK_E-cadherinΔE2-5GFP_) or E-cadherinΔE2-5GFP_ASAA_ (MDCK_E-cadherinΔE2-5GFPASAA)_. Here indeed, we found that E-cadherinΔE2-5GFP was enriched in EVs, whereas the quantities of E-cadherinΔE2-5GFP_ASAA_ were drastically reduced in isolated EVs (Figure 5D). Evidence for a central role of the cytosolic E-cadherin PTAP motif in Tsg101-interaction was provided by diminished Tsg101 precipitation of E-cadherinΔE2-5GFP_ASAA_ (Figure 5E). In conclusion, the PTAP motif in the cytoplasmic tail is essential for efficient Tsg101 interaction and EV-mediated release of E-cadherin.

**FIGURE 6:**
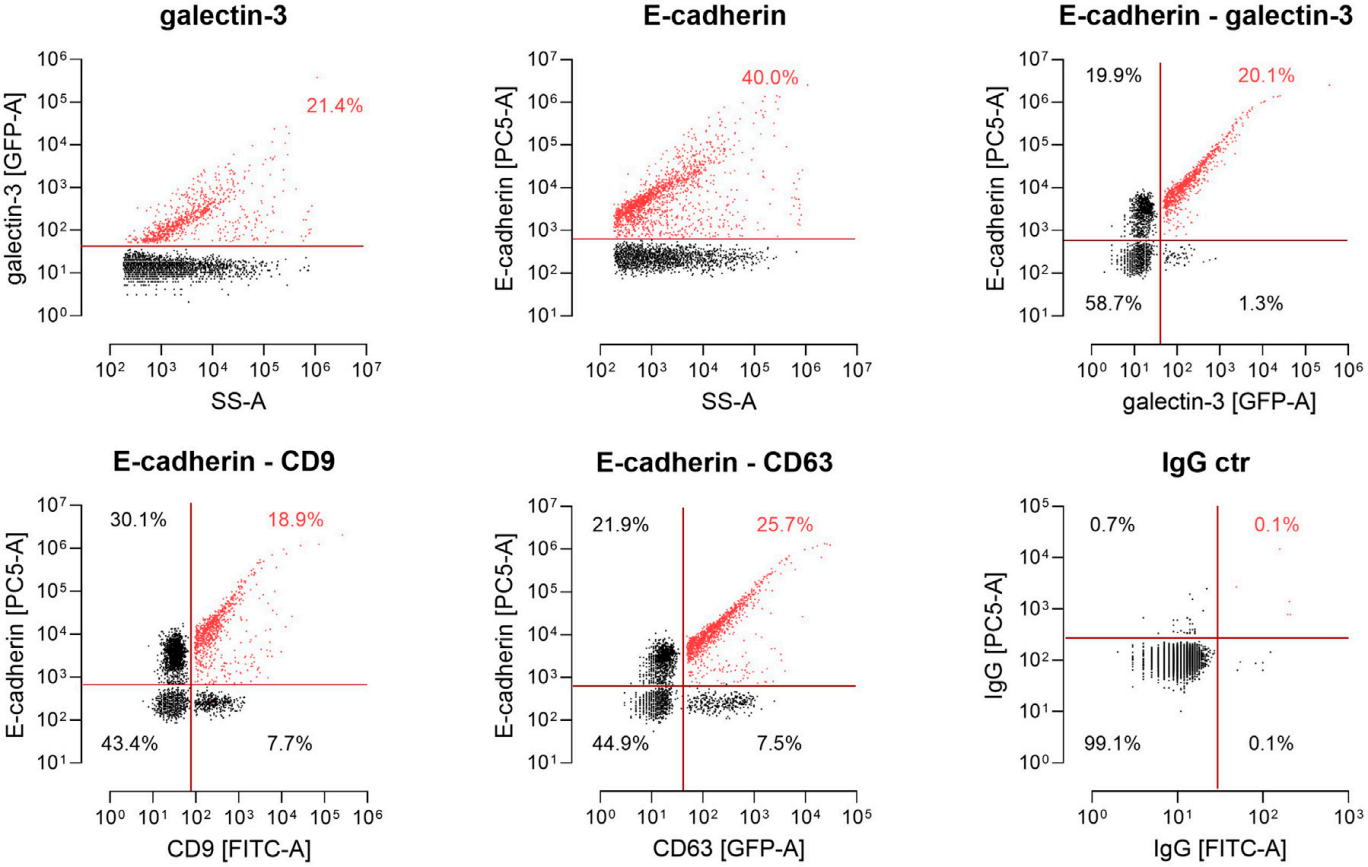
Single-particle phenotyping of EVs derived from MDCK_Gal3-GFP_ cells. Representative plots of galectin-3-GFP and E-cadherin (Ecad) expression on single EVs using the GFP signal and PE-conjugated antibodies by nano-flow cytometry (nFCM).Bivariate dot-plots of indicated fluorescence versus side scatter (SS-A). In addition, EVs harboring a CD63-GFP or fluorescently labeled with FITC-conjugated antibodies specific to CD9 were stained with PE-conjugated antibodies specific to Ecad. For E-cadherin-detection on vesicle surfaces, the Genetex/Biozol antibody GTX134997 was used. Double positives for CD9/Ecad and CD63/Ecad are depicted. Fluorescently labeled IgG isotypes were used as a control. Numbers indicate events detected in the corresponding gate in percent of total events.

Finally, we monitored the spectrum of EV populations released by MDCK cells using nano-flow cytometry (nFCM). Therefore, EVs were collected from the supernatants of MDCK or MDCK_Gal3-GFP_ cells and exposed polypeptides were fluorescently stained with antibodies directed against CD9, CD63 or E-cadherin to discriminate between distinct EV cargoes by flow cytometry (Figure 6). EV analysis at single particle level then revealed a strong correlation between the presence of E-cadherin and galectin-3 on extracellular vesicles. Galectin-3 is recruited by PSAP-mediated sorting into EVs (Bänfer et al., 2018) and shows the highest overlap with E-cadherin on these vesicles. Nearly all galectin-3-positive vesicles contain E-cadherin (20.1 % versus 1.3%). However, not all E-cadherin-positive vesicles are also loaded with galectin-3, which can be explained by a cargo-specific EV loading efficiency and modulation of this process by additional cellular factors. Less stringent correlation was detected between vesicles positive for the tetraspanins CD9-or CD-63- and E-cadherin. Altogether, these observations indicate that significant quantities of E-cadherin and galectin-3 are sorted into identical extracellular vesicles.

## Discussion

EVs provide an additional exchange platform for intercellular and interorgan communication. We had previously shown that galectin-3, a soluble lectin, can be loaded by an ESCRT-I-mediated mechanism based on a P(S/T)AP late domain into ILVs of MVEs. This study suggests a similar recruitment of E-cadherin, a type I transmembrane protein, into ILV-membranes.

Generally, proteins are targeted to MVEs after they are endocytosed from the plasma membrane (Lakkaraju and Rodriguez-Boulan, 2008). Hrs interacts with Tsg101 and has been described as an adaptor for ubiquitin-independent endosomal sorting of interleukin-2 receptor beta from early to LAMP1-positive late endosomes resulting in degradation of the receptor (Yamashita et al., 2008). Similarly, the G protein-coupled protease-activated receptor-1 and the purinergic receptor P2Y1 both contain a YPX_3_L motif, to which the ESCRT-associated protein ALG-2 interacting protein X (ALIX) binds (Dores et al., 2012). Moreover, recruitment of activated epidermal growth factor receptor (EGFR) into ILVs requires action of the ESCRT machinery (Katzmann et al., 2001). Here, ubiquitination serves as a sorting signal for selective entry of endocytosed cargo into ILVs, which are going to be degraded following delivery to the lysosomal lumen. Sequestering of cargos destined for degradation or exoscytosis expands the fate of MVEs beyond lysosomal fusion and ILV degradation. Thus, EGFR can be sorted by Rab31 into CD63-positive MVEs to prevent its lysosomal degradation (Wei et al., 2021). In this case flotillins are engaged to drive EGFR-containing ILV formation, which also depends on cholesterol and ceramide within lipid raft microdomains. This clearly shows how tightly balanced and intertwined the trafficking scenarios in the endosomal membrane system are. The question how ILVs containing E-cadherin are sorted away from the degradation pathway into MVEs for EV-release has not been solved yet and remains to be clarified.

As mentioned above specific lipid species are sorted into MVEs. The unique, poorly degradable phospholipid lysobisphosphatidic acid (LBPA) accumulates on MVE membranes and interacts with ALIX (Kobayashi et al., 1999; Matsuo et al., 2004). ALIX may thus support LBPA-enrichment at sites of ILV formation. Evidence for the involvement of ceramids in the biogenesis of EVs comes from experiments using inhibitors of neutral sphingomyelinases (Trajkovic et al., 2008; Menck et al., 2017). Ceramide is capable to self-associate through hydrogen bonding and can induce the coalescence of small microdomains into larger domains, which promotes microdomain-induced budding (Gulbins and Kolesnick, 2003). In polarized cells ceramides predominantly mediate the release of EVs from the basolateral membrane domain (Matsui et al., 2021). This study also describes apically secreted EVs that are formed in the presence of ALIX but independently of other ESCRT components. Together with our observation that the formation of galectin-3- as well as E-cadherin-positive EVs correlates with the presence of Tsg101, these data suggest that at least three distinct molecular mechanisms for the recruitment of EV cargo exist in epithelial cells, with one of them using the canonical ESCRT pathway. Consequently, this would lead to EV subpopulations composed of individual protein pools, which are formed in separate MVEs. Our nFCM-data point into the same direction. This idea of discrete EV biogenesis in individual endosomal compartments is confirmed by the finding that MVEs positive for the EV components CD9 or CD63 are stained separately in the cytoplasm of MDCK cells (Matsui et al., 2021). Release of specific EV subpopulations would then expand the extracellular vesicle bouquet and thereby enhance the spectrum of vesicle-mediated cell-cell communication in a living organism.

Two fates for E-cadherin on the EV membrane are plausible, it can remain intact or be cleaved of as soluble E-cadherin (Tang et al., 2018). Here, we found intact E-cadherin on isolated EVs, which would be more advantageous for long distance communication. EVs are extremely stable in human body fluids (Kalra et al., 2013) and thus provide a membrane environment that helps to increase the half-life of E-cadherin. Questions remain on the functional role of E-cadherin exposed on the EV membrane. Tang et al. (2018) reported that E-cadherin-positive EVs secreted from ovarian cells can promote angiogenesis. The cadherin heterogeneously interacts with VE-cadherin on the surface of endothelial cells. VE-mediated signaling then leads to increased nuclear accumulation of β-catenin and activation of the NFκB signaling cascade to induce angiogenesis. On the other hand, Zhang et al. (2020) claimed that EVs carrying E-cadherin promote the migration and invasion of adenocarcinomic human alveolar basal epithelial cells. They isolated EVs from the bronchoalveolar lavage fluid from patients with lung cancer. Release of E-cadherin positive EVs increases the E-cadherin concentration within the tumor microenvironment, thus facilitating lung cancer metastasis. Both analysis of E-cadherin-positive EVs and their functional effects are related to cancer progression. Moreover, in bone marrow dendritic cells E-cadherin is required to mediate the release of β-catenin into EVs (Chairoungdua et al., 2010). EV discharge of β-catenin might suppress tumor metastasis through down-regulation of the Wnt signaling pathway. This is an interesting observation, since interaction with E-cadherin is part of a process to recruit β-catenin into EVs. Considering these heterogenous examples of E-cadherin function on EVs, it remains to define in future studies regulatory elements that modulate formation and release of E-cadherin-positive EVs also under non-pathologic conditions.

## Data Availability

All datasets generated for this study are included in the article and/or the Supplementary Material.
