# Supplementary material for: Late domain dependent E-cadherin recruitment into extracellular vesicles
Source: Front Cell Dev Biol. 2022 Sep 7;10:878620. doi: 10.3389/fcell.2022.878620 (PMC9511404; doi:10.3389/fcell.2022.878620)
Supplement: Supplementary file 1 [file DataSheet1.PDF]

Hits for USERPAT1{P-S-A-P}, USERPAT2{P-T-A-P} motifs on all UniProtKB/Swiss-Prot (release 2018_09 of 10-Oct-18: 558590 entries) database sequences :
Graphical view (graphical view with feature detection)

| protein ID | from | to | USERPAT | motif | EXOCARTA-Hit |
|---|---|---|---|---|---|
| sp\|P78314\|3BP2_HUMAN | 286 | 289 | USERPAT2 | PTAP | |
| sp\|O60516\|4EBP3_HUMAN | 71 | 74 | USERPAT2 | PTAP | |
| sp\|Q8WXA8\|5HT3C_HUMAN | 362 | 365 | USERPAT2 | PTAP | |
| sp\|A5X5Y0\|5HT3E_HUMAN | 362 | 365 | USERPAT2 | PTAP | |
| sp\|P50406\|5HT6R_HUMAN | 20 | 23 | USERPAT1 | PSAP | |
| sp\|P08697\|A2AP_HUMAN | 438 | 441 | USERPAT1 | PSAP | |
| sp\|Q5T5F5\|A4AS1_HUMAN | 14 | 17 | USERPAT1 | PSAP | |
| sp\|P08910\|ABHD2_HUMAN | 38 | 41 | USERPAT2 | PTAP | |
| sp\|Q8WU67\|ABHD3_HUMAN | 210 | 213 | USERPAT1 | PSAP | |
| sp\|P12821\|ACE_HUMAN | 899 | 902 | USERPAT1 | PSAP | X |
| sp\|P78536\|ADA17_HUMAN | 746 | 749 | USERPAT1 | PSAP | X |
| sp\|O75077\|ADA23_HUMAN | 70 | 73 | USERPAT1 | PSAP | |
| sp\|Q6NVV9-7\|ADAM5_HUMAN | 402 | 405 | USERPAT2 | PTAP | |
| sp\|P78325\|ADAM8_HUMAN | 821 | 824 | USERPAT2 | PTAP | |
| sp\|Q6IQ32\|ADNP2_HUMAN | 283 | 286 | USERPAT1 | PSAP | |
| sp\|Q16186\|ADRM1_HUMAN | 231 | 234 | USERPAT1 | PSAP | X |
| sp\|P55198\|AF17_HUMAN | 372 | 375 | USERPAT2 | PTAP | |
| sp\|P51825\|AFF1_HUMAN | 451 | 454 | USERPAT1 | PSAP | |
| sp\|P51816\|AFF2_HUMAN | 132 | 135 | USERPAT1 | PSAP | |
| sp\|Q9UHB7\|AFF4_HUMAN | 891 | 894 | USERPAT1 | PSAP | X |
| sp\|O14514\|AGRB1_HUMAN | 129 | 132 | USERPAT1 | PSAP | |
| sp\|Q8IZP9\|AGRG2_HUMAN | 306 | 309 | USERPAT1 | PSAP | X |
| sp\|O00468\|AGRIN_HUMAN | 1043 | 1046 | USERPAT2 | PTAP | X |
| sp\|O95490\|AGRL2_HUMAN | 1200 | 1203 | USERPAT1 | PSAP | X |
| sp\|Q9NZN9\|AIPL1_HUMAN | 355 | 358 | USERPAT2 | PTAP | |
| sp\|Q96IF1\|AJUBA_HUMAN | 113 | 116 | USERPAT2 | PTAP | |
| sp\|Q5JQC9\|AKAP4_HUMAN | 191 | 194 | USERPAT1 | PSAP | |
| sp\|Q99996-6\|AKAP9_HUMAN | 3915 | 3918 | USERPAT2 | PTAP | X |
| sp\|Q495B1-1\|AKD1A_HUMAN | 505 | 508 | USERPAT1 | PSAP | |
| sp\|Q12802\|AKP13_HUMAN | 2710 | 2713 | USERPAT1 | PSAP | X |
| sp\|Q8TCU4\|ALMS1_HUMAN | 901 | 904 | USERPAT1 | PSAP | |
| sp\|Q86TB3\|ALPK2_HUMAN | 452 | 455 | USERPAT2 | PTAP | |
| sp\|P49418\|AMPH_HUMAN | 598 | 601 | USERPAT1 | PSAP | |
| sp\|Q400G9\|AMZ1_HUMAN | 219 | 222 | USERPAT1 | PSAP | |
| sp\|Q01484\|ANK2_HUMAN | 1047 | 1050 | USERPAT2 | PTAP | |
| sp\|Q12955\|ANK3_HUMAN | 2138 | 2141 | USERPAT1 | PSAP | X |
| sp\|P20594\|ANPRB_HUMAN | 144 | 147 | USERPAT1 | PSAP | |
| sp\|Q6UB99\|ANR11_HUMAN | 1749 | 1752 | USERPAT2 | PTAP | |
| sp\|Q9H6X2\|ANTR1_HUMAN | 526 | 529 | USERPAT1 | PSAP | X |
| sp\|O60641\|AP180_HUMAN | 810 | 813 | USERPAT1 | PSAP | |
| sp\|O43747\|AP1G1_HUMAN | 642 | 645 | USERPAT2 | PTAP | |
| sp\|O95996\|APCL_HUMAN | 1473 | 1476 | USERPAT1 | PSAP | |
| sp\|P05090\|APOD_HUMAN | 114 | 117 | USERPAT1 | PSAP | X |
| sp\|Q53RT3\|APRV1_HUMAN | 32 | 35 | USERPAT2 | PTAP | |
| sp\|A8MVX0\|ARG33_HUMAN | 502 | 505 | USERPAT1 | PSAP | |

| | | | | | |
|---|---|---|---|---|---|
| sp\|O15013\|ARHGA_HUMAN | 71 | 74 | USERPAT1 | PSAP | |
| sp\|O14497\|ARI1A_HUMAN | 1557 | 1560 | USERPAT1 | PSAP | X |
| sp\|Q8NFD5\|ARI1B_HUMAN | 443 | 446 | USERPAT1 | PSAP | X |
| sp\|Q8N7S6\|ARI2O_HUMAN | 100 | 103 | USERPAT2 | PTAP | |
| sp\|Q14865\|ARI5B_HUMAN | 543 | 546 | USERPAT1 | PSAP | X |
| sp\|Q8N5I2\|ARRD1_HUMAN | 309 | 312 | USERPAT1 | PSAP | |
| sp\|Q495Z4\|ASAS1_HUMAN | 5 | 8 | USERPAT1 | PSAP | |
| sp\|Q8WXI3-3\|ASB10_HUMAN | 19 | 22 | USERPAT1 | PSAP | X |
| sp\|Q99929\|ASCL2_HUMAN | 127 | 130 | USERPAT1 | PSAP | |
| sp\|Q9UHC3-3\|ASIC3_HUMAN | 528 | 531 | USERPAT2 | PTAP | X |
| sp\|Q5U4P2\|ASPH1_HUMAN | 196 | 199 | USERPAT1 | PSAP | X |
| sp\|Q96KQ4\|ASPP1_HUMAN | 777 | 780 | USERPAT2 | PTAP | |
| sp\|Q9C0F0\|ASXL3_HUMAN | 1572 | 1575 | USERPAT2 | PTAP | |
| sp\|Q4VNC1\|AT134_HUMAN | 1021 | 1024 | USERPAT2 | PTAP | |
| sp\|Q9ULK2\|AT7L1_HUMAN | 544 | 547 | USERPAT1 | PSAP | X |
| sp\|Q9Y2Q0\|AT8A1_HUMAN | 472 | 475 | USERPAT2 | PTAP | X |
| sp\|Q9NTI2\|AT8A2_HUMAN | 492 | 495 | USERPAT2 | PTAP | X |
| sp\|Q99941\|ATF6B_HUMAN | 420 | 423 | USERPAT1 | PSAP | |
| sp\|Q96BY7\|ATG2B_HUMAN | 970 | 973 | USERPAT2 | PTAP | |
| sp\|Q674R7\|ATG9B_HUMAN | 784 | 787 | USERPAT2 | PTAP | |
| sp\|P54259\|ATN1_HUMAN | 286 | 289 | USERPAT1 | PSAP | |
| sp\|Q96SQ7\|ATOH8_HUMAN | 165 | 168 | USERPAT1 | PSAP | |
| sp\|P54253\|ATX1_HUMAN | 90 | 93 | USERPAT1 | PSAP | X |
| sp\|O14965\|AURKA_HUMAN | 103 | 106 | USERPAT1 | PSAP | |
| sp\|Q13705\|AVR2B_HUMAN | 130 | 133 | USERPAT2 | PTAP | |
| sp\|O60909\|B4GT2_HUMAN | 89 | 92 | USERPAT2 | PTAP | |
| sp\|Q9BYV9\|BACH2_HUMAN | 320 | 323 | USERPAT2 | PTAP | X |
| sp\|O95429\|BAG4_HUMAN | 262 | 265 | USERPAT1 | PSAP | |
| sp\|P46379\|BAG6_HUMAN | 472 | 475 | USERPAT1 | PSAP | X |
| sp\|Q9P281\|BAHC1_HUMAN | 975 | 978 | USERPAT2 | PTAP | |
| sp\|Q9BZE3\|BARH1_HUMAN | 283 | 286 | USERPAT1 | PSAP | |
| sp\|Q9BXH1\|BBC3_HUMAN | 62 | 65 | USERPAT2 | PTAP | |
| sp\|O95999\|BCL10_HUMAN | 3 | 6 | USERPAT2 | PTAP | |
| sp\|Q8N143\|BCL6B_HUMAN | 153 | 156 | USERPAT2 | PTAP | |
| sp\|Q86UU0\|BCL9L_HUMAN | 455 | 458 | USERPAT2 | PTAP | |
| sp\|Q5H9F3\|BCORL_HUMAN | 323 | 326 | USERPAT1 | PSAP | |
| sp\|Q8NFC6\|BD1L1_HUMAN | 2195 | 2198 | USERPAT1 | PSAP | |
| sp\|O76090\|BEST1_HUMAN | 483 | 486 | USERPAT1 | PSAP | |
| sp\|Q7RTU4\|BHA09_HUMAN | 171 | 174 | USERPAT1 | PSAP | |
| sp\|Q9NZM4\|BICRA_HUMAN | 852 | 855 | USERPAT2 | PTAP | |
| sp\|Q9BWV1\|BOC_HUMAN | 692 | 695 | USERPAT1 | PSAP | |
| sp\|Q96GS4\|BORC6_HUMAN | 235 | 238 | USERPAT1 | PSAP | |
| sp\|P15056\|BRAF_HUMAN | 318 | 321 | USERPAT1 | PSAP | X |
| sp\|P59051\|BRAS2_HUMAN | 48 | 51 | USERPAT1 | PSAP | |
| sp\|O95696\|BRD1_HUMAN | 130 | 133 | USERPAT1 | PSAP | X |
| sp\|Q9UPA5\|BSN_HUMAN | 37 | 40 | USERPAT1 | PSAP | |
| sp\|Q96KE9\|BTBD6_HUMAN | 35 | 38 | USERPAT1 | PSAP | |
| sp\|Q5T0F9\|C2D1B_HUMAN | 350 | 353 | USERPAT2 | PTAP | |
| sp\|Q6ZS94\|CA229_HUMAN | 153 | 156 | USERPAT1 | PSAP | |
| sp\|Q9Y6J0\|CABIN_HUMAN | 1945 | 1948 | USERPAT2 | PTAP | |

| | | | | | |
|---|---|---|---|---|---|
| sp\|O43497\|CAC1G_HUMAN | 1964 | 1967 | USERPAT1 | PSAP | |
| sp\|Q9Y6N8\|CAD10_HUMAN | 702 | 705 | USERPAT2 | PTAP | |
| sp\|P55287\|CAD11_HUMAN | 732 | 735 | USERPAT2 | PTAP | |
| sp\|P55289\|CAD12_HUMAN | 728 | 731 | USERPAT2 | PTAP | |
| sp\|P12830\|CADH1_HUMAN | 822 | 825 | USERPAT2 | PTAP | X |
| sp\|P19022\|CADH2_HUMAN | 847 | 850 | USERPAT2 | PTAP | X |
| sp\|P22223\|CADH3_HUMAN | 769 | 772 | USERPAT2 | PTAP | X |
| sp\|P55283\|CADH4_HUMAN | 857 | 860 | USERPAT2 | PTAP | |
| sp\|P55285\|CADH6_HUMAN | 727 | 730 | USERPAT2 | PTAP | X |
| sp\|P55286\|CADH8_HUMAN | 736 | 739 | USERPAT2 | PTAP | |
| sp\|Q9ULB4\|CADH9_HUMAN | 727 | 730 | USERPAT1 | PSAP | |
| sp\|Q13111\|CAF1A_HUMAN | 846 | 849 | USERPAT1 | PSAP | |
| sp\|Q08AD1\|CAMP2_HUMAN | 1115 | 1118 | USERPAT2 | PTAP | |
| sp\|P55210-2\|CASP7_HUMAN | 233 | 236 | USERPAT1 | PSAP | |
| sp\|Q86V15\|CASZ1_HUMAN | 404 | 407 | USERPAT1 | PSAP | |
| sp\|Q8WUQ7-2\|CATIN_HUMAN | 892 | 895 | USERPAT2 | PTAP | |
| sp\|O95503\|CBX6_HUMAN | 302 | 305 | USERPAT1 | PSAP | |
| sp\|P0DN24\|CC086_HUMAN | 89 | 92 | USERPAT1 | PSAP | |
| sp\|Q8TBZ0\|CC110_HUMAN | 205 | 208 | USERPAT2 | PTAP | |
| sp\|P13994\|CC130_HUMAN | 257 | 260 | USERPAT1 | PSAP | |
| sp\|Q17RM4\|CC142_HUMAN | 542 | 545 | USERPAT1 | PSAP | |
| sp\|Q8NDH2\|CC168_HUMAN | 928 | 931 | USERPAT1 | PSAP | |
| sp\|Q9H0W5\|CCDC8_HUMAN | 164 | 167 | USERPAT1 | PSAP | |
| sp\|Q8WXS5\|CCG8_HUMAN | 398 | 401 | USERPAT1 | PSAP | |
| sp\|P53701\|CCHL_HUMAN | 5 | 8 | USERPAT1 | PSAP | |
| sp\|P14635\|CCNB1_HUMAN | 28 | 31 | USERPAT2 | PTAP | X |
| sp\|P08571\|CD14_HUMAN | 267 | 270 | USERPAT1 | PSAP | X |
| sp\|P06127\|CD5_HUMAN | 153 | 156 | USERPAT2 | PTAP | X |
| sp\|Q12834\|CDC20_HUMAN | 337 | 340 | USERPAT1 | PSAP | |
| sp\|Q9BYE9\|CDHR2_HUMAN | 1203 | 1206 | USERPAT1 | PSAP | X |
| sp\|Q9H305\|CDIP1_HUMAN | 13 | 16 | USERPAT2 | PTAP | |
| sp\|Q9NYV4\|CDK12_HUMAN | 1183 | 1186 | USERPAT1 | PSAP | X |
| sp\|Q9BWU1\|CDK19_HUMAN | 382 | 385 | USERPAT2 | PTAP | |
| sp\|P06731\|CEAM5_HUMAN | 4 | 7 | USERPAT1 | PSAP | X |
| sp\|P40199\|CEAM6_HUMAN | 4 | 7 | USERPAT1 | PSAP | |
| sp\|Q9HCU4\|CELR2_HUMAN | 1847 | 1850 | USERPAT1 | PSAP | |
| sp\|A0A0U1RR11\|CENL1_HUMAN | 77 | 80 | USERPAT1 | PSAP | |
| sp\|P0DPI3\|CENL2_HUMAN | 77 | 80 | USERPAT1 | PSAP | |
| sp\|A0A0U1RRI6\|CENL3_HUMAN | 92 | 95 | USERPAT1 | PSAP | |
| sp\|P49454\|CENPF_HUMAN | 1638 | 1641 | USERPAT1 | PSAP | X |
| sp\|Q5SRN2\|CF010_HUMAN | 175 | 178 | USERPAT1 | PSAP | |
| sp\|P0C671\|CF222_HUMAN | 40 | 43 | USERPAT2 | PTAP | |
| sp\|Q8NAV2\|CH058_HUMAN | 125 | 128 | USERPAT2 | PTAP | |
| sp\|Q8TD26\|CHD6_HUMAN | 2688 | 2691 | USERPAT1 | PSAP | |
| sp\|Q9H9P2\|CHODL_HUMAN | 186 | 189 | USERPAT2 | PTAP | |
| sp\|Q6ZV77\|CI139_HUMAN | 119 | 122 | USERPAT1 | PSAP | |
| sp\|Q96RK0\|CIC_HUMAN | 648 | 651 | USERPAT1 | PSAP | |
| sp\|Q9NPC3\|CIP1_HUMAN | 243 | 246 | USERPAT2 | PTAP | |
| sp\|Q5T1B1\|CJ091_HUMAN | 87 | 90 | USERPAT1 | PSAP | |
| sp\|Q7Z460\|CLAP1_HUMAN | 1149 | 1152 | USERPAT2 | PTAP | X |

| | | | | | |
|---|---|---|---|---|---|
| sp\|Q96DZ5\|CLIP3_HUMAN | 45 | 48 | USERPAT1 | PSAP | |
| sp\|Q96JQ2\|CLMN_HUMAN | 643 | 646 | USERPAT1 | PSAP | |
| sp\|Q9HAW4\|CLSPN_HUMAN | 1274 | 1277 | USERPAT1 | PSAP | |
| sp\|O94983\|CMTA2_HUMAN | 364 | 367 | USERPAT1 | PSAP | |
| sp\|Q8WXQ3\|CN183_HUMAN | 273 | 276 | USERPAT2 | PTAP | |
| sp\|O95628\|CNOT4_HUMAN | 365 | 368 | USERPAT2 | PTAP | X |
| sp\|P21554-2\|CNR1_HUMAN | 7 | 10 | USERPAT1 | PSAP | |
| sp\|P26992\|CNTFR_HUMAN | 346 | 349 | USERPAT1 | PSAP | X |
| sp\|Q6ZRI6\|CO039_HUMAN | 346 | 349 | USERPAT1 | PSAP | |
| sp\|P02461\|CO3A1_HUMAN | 92 | 95 | USERPAT2 | PTAP | |
| sp\|Q8IZC6\|CORA1_HUMAN | 372 | 375 | USERPAT1 | PSAP | |
| sp\|P57737\|CORO7_HUMAN | 115 | 118 | USERPAT1 | PSAP | |
| sp\|Q6UWD8\|CP054_HUMAN | 127 | 130 | USERPAT1 | PSAP | |
| sp\|P20813\|CP2B6_HUMAN | 258 | 261 | USERPAT1 | PSAP | |
| sp\|Q8N3J3\|CQ053_HUMAN | 65 | 68 | USERPAT2 | PTAP | |
| sp\|Q86X59\|CQ082_HUMAN | 58 | 61 | USERPAT1 | PSAP | |
| sp\|P20023\|CR2_HUMAN | 401 | 404 | USERPAT1 | PSAP | X |
| sp\|O43186\|CRX_HUMAN | 33 | 36 | USERPAT1 | PSAP | |
| sp\|P15509\|CSF2R_HUMAN | 142 | 145 | USERPAT2 | PTAP | |
| sp\|Q8WXD9\|CSKI1_HUMAN | 368 | 371 | USERPAT1 | PSAP | |
| sp\|Q8WXE0\|CSKI2_HUMAN | 357 | 360 | USERPAT1 | PSAP | |
| sp\|O95196\|CSPG5_HUMAN | 516 | 519 | USERPAT1 | PSAP | X |
| sp\|Q53GD3\|CTL4_HUMAN | 173 | 176 | USERPAT1 | PSAP | |
| sp\|O14529\|CUX2_HUMAN | 517 | 520 | USERPAT2 | PTAP | X |
| sp\|P17302\|CXA1_HUMAN | 274 | 277 | USERPAT2 | PTAP | |
| sp\|P57773\|CXA9_HUMAN | 279 | 282 | USERPAT1 | PSAP | |
| sp\|P36383\|CXG1_HUMAN | 284 | 287 | USERPAT1 | PSAP | |
| sp\|Q9H1C7\|CYTM1_HUMAN | 14 | 17 | USERPAT2 | PTAP | X |
| sp\|Q96B18\|DACT3_HUMAN | 171 | 174 | USERPAT1 | PSAP | |
| sp\|Q9UN19\|DAPP1_HUMAN | 159 | 162 | USERPAT2 | PTAP | X |
| sp\|Q8NFT6-3\|DBF4B_HUMAN | 412 | 415 | USERPAT2 | PTAP | X |
| sp\|Q9UK59\|DBR1_HUMAN | 273 | 276 | USERPAT1 | PSAP | |
| sp\|Q6ZNG2\|DBX2_HUMAN | 131 | 134 | USERPAT1 | PSAP | |
| sp\|Q9Y4B6\|DCAF1_HUMAN | 924 | 927 | USERPAT1 | PSAP | |
| sp\|Q96JK2\|DCAF5_HUMAN | 599 | 602 | USERPAT1 | PSAP | |
| sp\|P43146\|DCC_HUMAN | 429 | 432 | USERPAT1 | PSAP | |
| sp\|A8MYV0\|DCD2C_HUMAN | 6 | 9 | USERPAT1 | PSAP | |
| sp\|Q8TEH3\|DEN1A_HUMAN | 964 | 967 | USERPAT1 | PSAP | |
| sp\|O60231\|DHX16_HUMAN | 377 | 380 | USERPAT1 | PSAP | X |
| sp\|Q9UBP4\|DKK3_HUMAN | 18 | 21 | USERPAT2 | PTAP | X |
| sp\|Q9Y238-3\|DLEC1_HUMAN | 1720 | 1723 | USERPAT1 | PSAP | |
| sp\|Q8IXT2-2\|DMRTD_HUMAN | 158 | 161 | USERPAT1 | PSAP | |
| sp\|Q5JSL3\|DOC11_HUMAN | 925 | 928 | USERPAT1 | PSAP | |
| sp\|P49005\|DPOD2_HUMAN | 376 | 379 | USERPAT2 | PTAP | |
| sp\|Q07864\|DPOE1_HUMAN | 1207 | 1210 | USERPAT1 | PSAP | |
| sp\|Q8IXS2-2\|DRC2_HUMAN | 481 | 484 | USERPAT1 | PSAP | |
| sp\|Q8TD84\|DSCL1_HUMAN | 1996 | 1999 | USERPAT2 | PTAP | |
| sp\|Q14204\|DYHC1_HUMAN | 2527 | 2530 | USERPAT2 | PTAP | |
| sp\|Q01094\|E2F1_HUMAN | 76 | 79 | USERPAT1 | PSAP | X |
| sp\|O00716\|E2F3_HUMAN | 86 | 89 | USERPAT1 | PSAP | |

| | | | | | |
|---|---|---|---|---|---|
| sp\|Q9UNE0\|EDAR_HUMAN | 215 | 218 | USERPAT1 | PSAP | |
| sp\|Q6P2E9\|EDC4_HUMAN | 828 | 831 | USERPAT2 | PTAP | X |
| sp\|Q9BSW2-2\|EFC4B_HUMAN | 539 | 542 | USERPAT1 | PSAP | |
| sp\|Q9HA90\|EFCC1_HUMAN | 580 | 583 | USERPAT1 | PSAP | |
| sp\|Q96KS0\|EGLN2_HUMAN | 171 | 174 | USERPAT1 | PSAP | |
| sp\|Q9NVF9\|EKI2_HUMAN | 5 | 8 | USERPAT1 | PSAP | |
| sp\|Q8NFI3-3\|ENASE_HUMAN | 373 | 376 | USERPAT1 | PSAP | |
| sp\|B3EWF7\|EP2A2_HUMAN | 256 | 259 | USERPAT2 | PTAP | |
| sp\|Q09472\|EP300_HUMAN | 927 | 930 | USERPAT2 | PTAP | X |
| sp\|Q96L91\|EP400_HUMAN | 48 | 51 | USERPAT1 | PSAP | |
| sp\|P16452\|EPB42_HUMAN | 351 | 354 | USERPAT1 | PSAP | X |
| sp\|P21709\|EPHA1_HUMAN | 333 | 336 | USERPAT1 | PSAP | X |
| sp\|P29317\|EPHA2_HUMAN | 329 | 332 | USERPAT1 | PSAP | X |
| sp\|P54764\|EPHA4_HUMAN | 329 | 332 | USERPAT1 | PSAP | X |
| sp\|P54756\|EPHA5_HUMAN | 358 | 361 | USERPAT1 | PSAP | X |
| sp\|Q9UF33\|EPHA6_HUMAN | 332 | 335 | USERPAT1 | PSAP | |
| sp\|Q15375\|EPHA7_HUMAN | 332 | 335 | USERPAT1 | PSAP | |
| sp\|P29322\|EPHA8_HUMAN | 329 | 332 | USERPAT1 | PSAP | |
| sp\|Q5JZY3\|EPHAA_HUMAN | 338 | 341 | USERPAT1 | PSAP | |
| sp\|P54762-6\|EPHB1_HUMAN | 174 | 177 | USERPAT2 | PTAP | X |
| sp\|P29323\|EPHB2_HUMAN | 325 | 328 | USERPAT1 | PSAP | X |
| sp\|P54760\|EPHB4_HUMAN | 324 | 327 | USERPAT1 | PSAP | X |
| sp\|O15197\|EPHB6_HUMAN | 370 | 373 | USERPAT1 | PSAP | |
| sp\|Q5T890\|ER6L2_HUMAN | 14 | 17 | USERPAT1 | PSAP | |
| sp\|P04626-6\|ERBB2_HUMAN | 818 | 821 | USERPAT1 | PSAP | X |
| sp\|Q76MJ5\|ERN2_HUMAN | 770 | 773 | USERPAT1 | PSAP | |
| sp\|P62508\|ERR3_HUMAN | 86 | 89 | USERPAT1 | PSAP | |
| sp\|Q14674\|ESPL1_HUMAN | 1563 | 1566 | USERPAT1 | PSAP | |
| sp\|B1AK53\|ESPN_HUMAN | 567 | 570 | USERPAT1 | PSAP | |
| sp\|Q92731\|ESR2_HUMAN | 285 | 288 | USERPAT1 | PSAP | |
| sp\|Q9BSJ8\|ESYT1_HUMAN | 17 | 20 | USERPAT1 | PSAP | X |
| sp\|P41162\|ETV3_HUMAN | 459 | 462 | USERPAT1 | PSAP | |
| sp\|Q03828\|EVX2_HUMAN | 54 | 57 | USERPAT1 | PSAP | |
| sp\|Q01844\|EWS_HUMAN | 80 | 83 | USERPAT2 | PTAP | |
| sp\|Q9BQ89\|F110A_HUMAN | 11 | 14 | USERPAT1 | PSAP | |
| sp\|Q6P1L5\|F117B_HUMAN | 193 | 196 | USERPAT1 | PSAP | |
| sp\|Q9P2D6\|F135A_HUMAN | 1156 | 1159 | USERPAT1 | PSAP | |
| sp\|O75949\|F155B_HUMAN | 107 | 110 | USERPAT1 | PSAP | |
| sp\|Q8N612\|F16A2_HUMAN | 87 | 90 | USERPAT1 | PSAP | |
| sp\|Q86V87\|F16B2_HUMAN | 324 | 327 | USERPAT1 | PSAP | |
| sp\|A6NEQ2\|F181B_HUMAN | 132 | 135 | USERPAT1 | PSAP | |
| sp\|A6NE01\|F186A_HUMAN | 1971 | 1974 | USERPAT1 | PSAP | |
| sp\|Q9NTX9\|F217B_HUMAN | 209 | 212 | USERPAT2 | PTAP | |
| sp\|Q96D05-2\|F241B_HUMAN | 37 | 40 | USERPAT2 | PTAP | |
| sp\|Q8N5Q1\|F71E2_HUMAN | 522 | 525 | USERPAT2 | PTAP | |
| sp\|A6NJQ4\|F90A8_HUMAN | 30 | 33 | USERPAT1 | PSAP | |
| sp\|Q14153\|FA53B_HUMAN | 105 | 108 | USERPAT1 | PSAP | |
| sp\|A6ND36\|FA83G_HUMAN | 480 | 483 | USERPAT1 | PSAP | |
| sp\|Q9UBU6\|FA8A1_HUMAN | 53 | 56 | USERPAT2 | PTAP | |
| sp\|Q6V0I7\|FAT4_HUMAN | 4606 | 4609 | USERPAT1 | PSAP | X |

| | | | | | |
|---|---|---|---|---|---|
| sp\|Q6P3S6\|FBX42_HUMAN | 468 | 471 | USERPAT1 | PSAP | |
| sp\|P06734\|FCER2_HUMAN | 315 | 318 | USERPAT1 | PSAP | X |
| sp\|Q0JRZ9\|FCHO2_HUMAN | 412 | 415 | USERPAT1 | PSAP | |
| sp\|Q6DN72\|FCRL6_HUMAN | 345 | 348 | USERPAT2 | PTAP | |
| sp\|A0PJY2\|FEZF1_HUMAN | 110 | 113 | USERPAT1 | PSAP | |
| sp\|P08620\|FGF4_HUMAN | 32 | 35 | USERPAT2 | PTAP | |
| sp\|A6NMB9\|FIGL2_HUMAN | 198 | 201 | USERPAT1 | PSAP | |
| sp\|Q4L180\|FIL1L_HUMAN | 1083 | 1086 | USERPAT1 | PSAP | |
| sp\|Q14318\|FKBP8_HUMAN | 10 | 13 | USERPAT1 | PSAP | X |
| sp\|Q14315\|FLNC_HUMAN | 1920 | 1923 | USERPAT2 | PTAP | X |
| sp\|P49771\|FLT3L_HUMAN | 179 | 182 | USERPAT2 | PTAP | |
| sp\|O95466\|FMNL1_HUMAN | 559 | 562 | USERPAT1 | PSAP | |
| sp\|Q96PY5\|FMNL2_HUMAN | 592 | 595 | USERPAT1 | PSAP | X |
| sp\|Q53EP0\|FND3B_HUMAN | 478 | 481 | USERPAT1 | PSAP | |
| sp\|Q4ZHG4\|FNDC1_HUMAN | 1622 | 1625 | USERPAT1 | PSAP | X |
| sp\|Q8NAU1\|FNDC5_HUMAN | 34 | 37 | USERPAT1 | PSAP | |
| sp\|Q5VYV0\|FOXB2_HUMAN | 185 | 188 | USERPAT2 | PTAP | |
| sp\|Q12948\|FOXC1_HUMAN | 297 | 300 | USERPAT1 | PSAP | |
| sp\|Q12947\|FOXF2_HUMAN | 231 | 234 | USERPAT1 | PSAP | |
| sp\|Q6ZQN5\|FOXI2_HUMAN | 236 | 239 | USERPAT1 | PSAP | |
| sp\|O15353\|FOXN1_HUMAN | 628 | 631 | USERPAT2 | PTAP | |
| sp\|A8MYZ6\|FOXO6_HUMAN | 216 | 219 | USERPAT1 | PSAP | |
| sp\|Q9H334\|FOXP1_HUMAN | 410 | 413 | USERPAT2 | PTAP | X |
| sp\|O15409\|FOXP2_HUMAN | 450 | 453 | USERPAT2 | PTAP | X |
| sp\|Q9BZS1\|FOXP3_HUMAN | 9 | 12 | USERPAT1 | PSAP | |
| sp\|Q9C009\|FOXQ1_HUMAN | 343 | 346 | USERPAT2 | PTAP | X |
| sp\|Q5JV73\|FRPD3_HUMAN | 1046 | 1049 | USERPAT1 | PSAP | |
| sp\|Q9P0K9\|FRS1L_HUMAN | 135 | 138 | USERPAT2 | PTAP | |
| sp\|Q9BTV5\|FSD1_HUMAN | 165 | 168 | USERPAT1 | PSAP | |
| sp\|A1L4K1\|FSD2_HUMAN | 372 | 375 | USERPAT1 | PSAP | X |
| sp\|Q13467\|FZD5_HUMAN | 6 | 9 | USERPAT1 | PSAP | X |
| sp\|O75084\|FZD7_HUMAN | 180 | 183 | USERPAT2 | PTAP | X |
| sp\|Q96RP7\|G3ST4_HUMAN | 51 | 54 | USERPAT1 | PSAP | |
| sp\|P62684\|GA113_HUMAN | 253 | 256 | USERPAT2 | PTAP | |
| sp\|Q9YNA8\|GAK19_HUMAN | 253 | 256 | USERPAT2 | PTAP | |
| sp\|P62683\|GAK21_HUMAN | 253 | 256 | USERPAT2 | PTAP | |
| sp\|P63145\|GAK24_HUMAN | 253 | 256 | USERPAT2 | PTAP | |
| sp\|Q7LDI9\|GAK6_HUMAN | 253 | 256 | USERPAT2 | PTAP | |
| sp\|P63130\|GAK7_HUMAN | 253 | 256 | USERPAT2 | PTAP | |
| sp\|P62685\|GAK8_HUMAN | 253 | 256 | USERPAT2 | PTAP | |
| sp\|P63126\|GAK9_HUMAN | 253 | 256 | USERPAT2 | PTAP | |
| sp\|Q92908\|GATA6_HUMAN | 94 | 97 | USERPAT1 | PSAP | |
| sp\|P48169\|GBRA4_HUMAN | 505 | 508 | USERPAT1 | PSAP | |
| sp\|Q96RT7\|GCP6_HUMAN | 931 | 934 | USERPAT1 | PSAP | |
| sp\|Q6KF10\|GDF6_HUMAN | 173 | 176 | USERPAT1 | PSAP | |
| sp\|Q9GZZ7-2\|GFRA4_HUMAN | 145 | 148 | USERPAT1 | PSAP | |
| sp\|I3L273\|GFY_HUMAN | 23 | 26 | USERPAT1 | PSAP | |
| sp\|Q9NZ52\|GGA3_HUMAN | 449 | 452 | USERPAT1 | PSAP | X |
| sp\|Q86UU5\|GGN_HUMAN | 326 | 329 | USERPAT1 | PSAP | |
| sp\|P19440\|GGT1_HUMAN | 277 | 280 | USERPAT1 | PSAP | X |

| | | | | | |
|---|---|---|---|---|---|
| sp\|P10070\|GLI2_HUMAN | 1119 | 1122 | USERPAT1 | PSAP | |
| sp\|Q8NEA6\|GLIS3_HUMAN | 614 | 617 | USERPAT1 | PSAP | |
| sp\|P14314\|GLU2B_HUMAN | 292 | 295 | USERPAT1 | PSAP | |
| sp\|Q5JWF2\|GNAS1_HUMAN | 497 | 500 | USERPAT2 | PTAP | X |
| sp\|Q9NVN8\|GNL3L_HUMAN | 41 | 44 | USERPAT1 | PSAP | |
| sp\|Q14439\|GP176_HUMAN | 420 | 423 | USERPAT1 | PSAP | |
| sp\|Q6PRD1\|GP179_HUMAN | 2268 | 2271 | USERPAT2 | PTAP | |
| sp\|Q9NZH0\|GPC5B_HUMAN | 367 | 370 | USERPAT1 | PSAP | |
| sp\|Q96P69\|GPR78_HUMAN | 73 | 76 | USERPAT1 | PSAP | |
| sp\|Q6IC98\|GRAM4_HUMAN | 424 | 427 | USERPAT1 | PSAP | |
| sp\|Q4V328-2\|GRAP1_HUMAN | 615 | 618 | USERPAT2 | PTAP | |
| sp\|Q4ZG55\|GREB1_HUMAN | 321 | 324 | USERPAT1 | PSAP | X |
| sp\|Q7LDG7\|GRP2_HUMAN | 215 | 218 | USERPAT2 | PTAP | |
| sp\|Q4G1C9-3\|GRPL2_HUMAN | 102 | 105 | USERPAT1 | PSAP | |
| sp\|O95528\|GTR10_HUMAN | 383 | 386 | USERPAT1 | PSAP | |
| sp\|Q5JVS0\|HABP4_HUMAN | 252 | 255 | USERPAT2 | PTAP | |
| sp\|Q9Y3Q4\|HCN4_HUMAN | 890 | 893 | USERPAT1 | PSAP | |
| sp\|Q9P2P5\|HECW2_HUMAN | 838 | 841 | USERPAT2 | PTAP | |
| sp\|A6NFD8\|HELT_HUMAN | 192 | 195 | USERPAT1 | PSAP | |
| sp\|O95714\|HERC2_HUMAN | 3486 | 3489 | USERPAT1 | PSAP | X |
| sp\|Q9Y543\|HES2_HUMAN | 139 | 142 | USERPAT1 | PSAP | |
| sp\|Q9Y5J3\|HEY1_HUMAN | 281 | 284 | USERPAT1 | PSAP | |
| sp\|O14964\|HGS_HUMAN | 348 | 351 | USERPAT1 | PSAP | X |
| sp\|Q9UM44\|HHLA2_HUMAN | 398 | 401 | USERPAT1 | PSAP | |
| sp\|O14979\|HNRDL_HUMAN | 16 | 19 | USERPAT1 | PSAP | |
| sp\|Q92902\|HPS1_HUMAN | 387 | 390 | USERPAT1 | PSAP | |
| sp\|Q9NQG7\|HPS4_HUMAN | 428 | 431 | USERPAT1 | PSAP | X |
| sp\|Q9Y278\|HS3S2_HUMAN | 83 | 86 | USERPAT1 | PSAP | |
| sp\|Q7Z6Z7\|HUWE1_HUMAN | 3505 | 3508 | USERPAT2 | PTAP | X |
| sp\|O43248\|HXC11_HUMAN | 127 | 130 | USERPAT1 | PSAP | |
| sp\|P35453\|HXD13_HUMAN | 120 | 123 | USERPAT1 | PSAP | |
| sp\|Q12891\|HYAL2_HUMAN | 25 | 28 | USERPAT2 | PTAP | X |
| sp\|Q6UWB1\|I27RA_HUMAN | 230 | 233 | USERPAT1 | PSAP | |
| sp\|Q9H1B7\|I2BPL_HUMAN | 518 | 521 | USERPAT1 | PSAP | |
| sp\|Q659A1\|ICE2_HUMAN | 432 | 435 | USERPAT2 | PTAP | |
| sp\|P35475-2\|IDUA_HUMAN | 223 | 226 | USERPAT1 | PSAP | |
| sp\|Q9BTL4\|IER2_HUMAN | 194 | 197 | USERPAT2 | PTAP | X |
| sp\|P23588\|IF4B_HUMAN | 74 | 77 | USERPAT2 | PTAP | |
| sp\|Q8TDY8\|IGDC4_HUMAN | 429 | 432 | USERPAT1 | PSAP | |
| sp\|Q86VF2\|IGFN1_HUMAN | 747 | 750 | USERPAT1 | PSAP | |
| sp\|Q5DX21\|IGS11_HUMAN | 144 | 147 | USERPAT1 | PSAP | |
| sp\|Q9BYH8\|IKBZ_HUMAN | 57 | 60 | USERPAT1 | PSAP | |
| sp\|Q14005\|IL16_HUMAN | 339 | 342 | USERPAT1 | PSAP | |
| sp\|C9JVW0\|INAM1_HUMAN | 69 | 72 | USERPAT1 | PSAP | |
| sp\|Q27J81\|INF2_HUMAN | 455 | 458 | USERPAT2 | PTAP | X |
| sp\|Q3MIP1\|IPIL2_HUMAN | 330 | 333 | USERPAT1 | PSAP | |
| sp\|Q8NFU5\|IPMK_HUMAN | 120 | 123 | USERPAT2 | PTAP | |
| sp\|Q4KMZ1\|IQCC_HUMAN | 224 | 227 | USERPAT1 | PSAP | |
| sp\|Q8WZA9\|IRGQ_HUMAN | 258 | 261 | USERPAT2 | PTAP | |
| sp\|P16144\|ITB4_HUMAN | 1641 | 1644 | USERPAT1 | PSAP | |

| | | | | | |
|---|---|---|---|---|---|
| sp\|Q6UXX5\|ITIH6_HUMAN | 858 | 861 | USERPAT1 | PSAP | |
| sp\|P28290\|ITPI2_HUMAN | 1157 | 1160 | USERPAT2 | PTAP | |
| sp\|Q13387\|JIP2_HUMAN | 496 | 499 | USERPAT1 | PSAP | |
| sp\|Q96MG2\|JSPR1_HUMAN | 185 | 188 | USERPAT1 | PSAP | |
| sp\|O15063\|K0355_HUMAN | 994 | 997 | USERPAT1 | PSAP | |
| sp\|Q6ZU52\|K0408_HUMAN | 670 | 673 | USERPAT1 | PSAP | |
| sp\|Q6ZU52-2\|K0408_HUMAN | 553 | 556 | USERPAT1 | PSAP | |
| sp\|O60303\|K0556_HUMAN | 712 | 715 | USERPAT1 | PSAP | |
| sp\|Q9P206\|K1522_HUMAN | 846 | 849 | USERPAT1 | PSAP | |
| sp\|Q9HCM3\|K1549_HUMAN | 1891 | 1894 | USERPAT1 | PSAP | |
| sp\|Q6ZVL6\|K154L_HUMAN | 443 | 446 | USERPAT2 | PTAP | |
| sp\|Q5VZ46\|K1614_HUMAN | 788 | 791 | USERPAT1 | PSAP | |
| sp\|Q9BY89\|K1671_HUMAN | 1301 | 1304 | USERPAT1 | PSAP | |
| sp\|P23352\|KALM_HUMAN | 287 | 290 | USERPAT1 | PSAP | |
| sp\|Q92830\|KAT2A_HUMAN | 129 | 132 | USERPAT2 | PTAP | |
| sp\|Q92993\|KAT5_HUMAN | 207 | 210 | USERPAT1 | PSAP | |
| sp\|Q96NX5\|KCC1G_HUMAN | 381 | 384 | USERPAT2 | PTAP | |
| sp\|Q9ULD8\|KCNH3_HUMAN | 797 | 800 | USERPAT1 | PSAP | |
| sp\|Q8N5I3\|KCNRG_HUMAN | 147 | 150 | USERPAT1 | PSAP | |
| sp\|P41229\|KDM5C_HUMAN | 1536 | 1539 | USERPAT1 | PSAP | X |
| sp\|O15054\|KDM6B_HUMAN | 1047 | 1050 | USERPAT2 | PTAP | X |
| sp\|Q5VWX1\|KHDR2_HUMAN | 199 | 202 | USERPAT2 | PTAP | |
| sp\|Q9NQT8\|KI13B_HUMAN | 1596 | 1599 | USERPAT2 | PTAP | X |
| sp\|Q96FN5\|KIF12_HUMAN | 583 | 586 | USERPAT1 | PSAP | X |
| sp\|Q96AC6\|KIFC2_HUMAN | 776 | 779 | USERPAT1 | PSAP | |
| sp\|Q8TD94\|KLF14_HUMAN | 151 | 154 | USERPAT1 | PSAP | |
| sp\|O43474\|KLF4_HUMAN | 181 | 184 | USERPAT2 | PTAP | X |
| sp\|Q9P0G3\|KLK14_HUMAN | 12 | 15 | USERPAT1 | PSAP | |
| sp\|Q03164\|KMT2A_HUMAN | 978 | 981 | USERPAT2 | PTAP | |
| sp\|O14686\|KMT2D_HUMAN | 2190 | 2193 | USERPAT2 | PTAP | X |
| sp\|Q76NI1-4\|KNDC1_HUMAN | 1351 | 1354 | USERPAT1 | PSAP | |
| sp\|Q93100\|KPBB_HUMAN | 703 | 706 | USERPAT1 | PSAP | |
| sp\|Q9BZL6\|KPCD2_HUMAN | 517 | 520 | USERPAT1 | PSAP | |
| sp\|Q9BVA0\|KTNB1_HUMAN | 329 | 332 | USERPAT1 | PSAP | |
| sp\|Q16787\|LAMA3_HUMAN | 88 | 91 | USERPAT2 | PTAP | X |
| sp\|O15230\|LAMA5_HUMAN | 1906 | 1909 | USERPAT1 | PSAP | |
| sp\|Q569H4\|LARGN_HUMAN | 286 | 289 | USERPAT2 | PTAP | |
| sp\|O43561\|LAT_HUMAN | 177 | 180 | USERPAT1 | PSAP | X |
| sp\|Q6UX15\|LAYN_HUMAN | 157 | 160 | USERPAT1 | PSAP | |
| sp\|P18428\|LBP_HUMAN | 344 | 347 | USERPAT1 | PSAP | X |
| sp\|Q6XYB7\|LBX2_HUMAN | 29 | 32 | USERPAT1 | PSAP | |
| sp\|Q8NC56\|LEMD2_HUMAN | 236 | 239 | USERPAT1 | PSAP | |
| sp\|Q96PV6\|LENG8_HUMAN | 131 | 134 | USERPAT1 | PSAP | |
| sp\|Q9BWQ8\|LFG2_HUMAN | 32 | 35 | USERPAT1 | PSAP | |
| sp\|Q969X1\|LFG3_HUMAN | 4 | 7 | USERPAT1 | PSAP | |
| sp\|Q13136\|LIPA1_HUMAN | 947 | 950 | USERPAT1 | PSAP | |
| sp\|O75334\|LIPA2_HUMAN | 967 | 970 | USERPAT1 | PSAP | |
| sp\|O75145\|LIPA3_HUMAN | 907 | 910 | USERPAT1 | PSAP | |
| sp\|O75335\|LIPA4_HUMAN | 898 | 901 | USERPAT1 | PSAP | |
| sp\|Q05469\|LIPS_HUMAN | 169 | 172 | USERPAT1 | PSAP | |

| | | | | | |
|---|---|---|---|---|---|
| sp\|Q99732\|LITAF_HUMAN | 17 | 20 | USERPAT1 | PSAP | |
| sp\|Q6ZMQ8\|LMTK1_HUMAN | 1274 | 1277 | USERPAT1 | PSAP | |
| sp\|Q1L5Z9\|LONF2_HUMAN | 392 | 395 | USERPAT2 | PTAP | |
| sp\|Q15345\|LRC41_HUMAN | 358 | 361 | USERPAT1 | PSAP | |
| sp\|Q9ULH4\|LRFN2_HUMAN | 644 | 647 | USERPAT1 | PSAP | |
| sp\|Q96JA1\|LRIG1_HUMAN | 959 | 962 | USERPAT1 | PSAP | |
| sp\|O75074\|LRP3_HUMAN | 668 | 671 | USERPAT1 | PSAP | |
| sp\|Q9BY71\|LRRC3_HUMAN | 244 | 247 | USERPAT1 | PSAP | |
| sp\|Q6UWE0\|LRSM1_HUMAN | 649 | 652 | USERPAT2 | PTAP | X |
| sp\|Q8ND56\|LS14A_HUMAN | 265 | 268 | USERPAT1 | PSAP | |
| sp\|O60449\|LY75_HUMAN | 307 | 310 | USERPAT1 | PSAP | X |
| sp\|P16109\|LYAM3_HUMAN | 139 | 142 | USERPAT1 | PSAP | |
| sp\|Q5VWZ2\|LYPL1_HUMAN | 59 | 62 | USERPAT2 | PTAP | X |
| sp\|Q8IV50\|LYSM2_HUMAN | 20 | 23 | USERPAT1 | PSAP | |
| sp\|Q99698\|LYST_HUMAN | 2578 | 2581 | USERPAT1 | PSAP | X |
| sp\|Q02779\|M3K10_HUMAN | 656 | 659 | USERPAT1 | PSAP | |
| sp\|Q5TCX8\|M3K21_HUMAN | 840 | 843 | USERPAT2 | PTAP | |
| sp\|Q99759\|M3K3_HUMAN | 356 | 359 | USERPAT1 | PSAP | |
| sp\|Q92918-2\|M4K1_HUMAN | 811 | 814 | USERPAT2 | PTAP | |
| sp\|Q3KQU3-3\|MA7D1_HUMAN | 114 | 117 | USERPAT2 | PTAP | |
| sp\|Q9HCI5\|MAGE1_HUMAN | 394 | 397 | USERPAT2 | PTAP | |
| sp\|Q9UJ55\|MAGL2_HUMAN | 132 | 135 | USERPAT1 | PSAP | |
| sp\|Q92585\|MAML1_HUMAN | 921 | 924 | USERPAT2 | PTAP | |
| sp\|Q16798\|MAON_HUMAN | 27 | 30 | USERPAT2 | PTAP | |
| sp\|P78559\|MAP1A_HUMAN | 1811 | 1814 | USERPAT1 | PSAP | X |
| sp\|Q66K74\|MAP1S_HUMAN | 708 | 711 | USERPAT1 | PSAP | X |
| sp\|Q96JE9\|MAP6_HUMAN | 801 | 804 | USERPAT2 | PTAP | |
| sp\|Q8NA82\|MARHA_HUMAN | 223 | 226 | USERPAT1 | PSAP | |
| sp\|Q6P0Q8\|MAST2_HUMAN | 1708 | 1711 | USERPAT1 | PSAP | |
| sp\|Q96DN6\|MBD6_HUMAN | 614 | 617 | USERPAT1 | PSAP | X |
| sp\|Q9NR56\|MBNL1_HUMAN | 151 | 154 | USERPAT2 | PTAP | X |
| sp\|Q6VMQ6\|MCAF1_HUMAN | 1031 | 1034 | USERPAT2 | PTAP | X |
| sp\|Q14676\|MDC1_HUMAN | 1163 | 1166 | USERPAT2 | PTAP | |
| sp\|Q03112\|MECOM_HUMAN | 482 | 485 | USERPAT2 | PTAP | X |
| sp\|Q93074\|MED12_HUMAN | 561 | 564 | USERPAT1 | PSAP | |
| sp\|O60244\|MED14_HUMAN | 25 | 28 | USERPAT1 | PSAP | |
| sp\|Q7Z7M0\|MEGF8_HUMAN | 621 | 624 | USERPAT2 | PTAP | X |
| sp\|Q7L2J0\|MEPCE_HUMAN | 286 | 289 | USERPAT2 | PTAP | X |
| sp\|Q14696\|MESD_HUMAN | 87 | 90 | USERPAT1 | PSAP | |
| sp\|A1L020\|MEX3A_HUMAN | 104 | 107 | USERPAT2 | PTAP | |
| sp\|Q8IWA4\|MFN1_HUMAN | 563 | 566 | USERPAT2 | PTAP | |
| sp\|A6NFX1\|MFS2B_HUMAN | 259 | 262 | USERPAT1 | PSAP | X |
| sp\|Q6ZSS7\|MFSD6_HUMAN | 215 | 218 | USERPAT2 | PTAP | X |
| sp\|O60291\|MGRN1_HUMAN | 406 | 409 | USERPAT1 | PSAP | X |
| sp\|Q8IY33\|MILK2_HUMAN | 366 | 369 | USERPAT1 | PSAP | |
| sp\|Q8IXI1\|MIRO2_HUMAN | 169 | 172 | USERPAT2 | PTAP | |
| sp\|Q8NDC0\|MISSL_HUMAN | 43 | 46 | USERPAT1 | PSAP | |
| sp\|Q13164\|MK07_HUMAN | 495 | 498 | USERPAT1 | PSAP | |
| sp\|Q9HAP2\|MLXIP_HUMAN | 386 | 389 | USERPAT2 | PTAP | |
| sp\|P51511\|MMP15_HUMAN | 5 | 8 | USERPAT1 | PSAP | X |

| | | | | | |
|---|---|---|---|---|---|
| sp\|Q8N119\|MMP21_HUMAN | 127 | 130 | USERPAT1 | PSAP | |
| sp\|P14780\|MMP9_HUMAN | 461 | 464 | USERPAT2 | PTAP | X |
| sp\|Q99583\|MNT_HUMAN | 472 | 475 | USERPAT1 | PSAP | |
| sp\|Q96EN8\|MOCOS_HUMAN | 21 | 24 | USERPAT1 | PSAP | |
| sp\|O60669\|MOT2_HUMAN | 5 | 8 | USERPAT1 | PSAP | |
| sp\|Q9ULH7\|MRTFB_HUMAN | 607 | 610 | USERPAT1 | PSAP | |
| sp\|O15457\|MSH4_HUMAN | 11 | 14 | USERPAT1 | PSAP | |
| sp\|O43347\|MSI1H_HUMAN | 254 | 257 | USERPAT1 | PSAP | |
| sp\|Q86U44\|MTA70_HUMAN | 53 | 56 | USERPAT2 | PTAP | |
| sp\|Q14872\|MTF1_HUMAN | 440 | 443 | USERPAT1 | PSAP | X |
| sp\|O95248\|MTMR5_HUMAN | 119 | 122 | USERPAT2 | PTAP | |
| sp\|Q8WXI7\|MUC16_HUMAN | 3877 | 3880 | USERPAT1 | PSAP | X |
| sp\|Q02817\|MUC2_HUMAN | 4256 | 4259 | USERPAT2 | PTAP | |
| sp\|P98088\|MUC5A_HUMAN | 3013 | 3016 | USERPAT1 | PSAP | X |
| sp\|Q9HC84\|MUC5B_HUMAN | 1495 | 1498 | USERPAT1 | PSAP | |
| sp\|Q6W4X9\|MUC6_HUMAN | 2135 | 2138 | USERPAT1 | PSAP | X |
| sp\|Q13203\|MYBPH_HUMAN | 71 | 74 | USERPAT1 | PSAP | |
| sp\|Q15746\|MYLK_HUMAN | 620 | 623 | USERPAT2 | PTAP | X |
| sp\|Q9NZM1\|MYOF_HUMAN | 127 | 130 | USERPAT1 | PSAP | X |
| sp\|P52179\|MYOM1_HUMAN | 617 | 620 | USERPAT1 | PSAP | |
| sp\|P54296\|MYOM2_HUMAN | 612 | 615 | USERPAT1 | PSAP | |
| sp\|Q5VTT5\|MYOM3_HUMAN | 700 | 703 | USERPAT1 | PSAP | |
| sp\|Q5VU43\|MYOME_HUMAN | 1712 | 1715 | USERPAT1 | PSAP | |
| sp\|Q93015\|NAA80_HUMAN | 213 | 216 | USERPAT2 | PTAP | |
| sp\|O15069\|NACAD_HUMAN | 1229 | 1232 | USERPAT1 | PSAP | |
| sp\|Q8N159\|NAGS_HUMAN | 180 | 183 | USERPAT2 | PTAP | |
| sp\|P59046\|NAL12_HUMAN | 275 | 278 | USERPAT1 | PSAP | |
| sp\|Q8IZF0\|NALCN_HUMAN | 1673 | 1676 | USERPAT1 | PSAP | |
| sp\|Q8IVL1\|NAV2_HUMAN | 600 | 603 | USERPAT1 | PSAP | |
| sp\|A0A0U1RRE5\|NBDY_HUMAN | 49 | 52 | USERPAT1 | PSAP | |
| sp\|O60934\|NBN_HUMAN | 354 | 357 | USERPAT1 | PSAP | |
| sp\|Q14919\|NC2A_HUMAN | 192 | 195 | USERPAT1 | PSAP | |
| sp\|P13591\|NCAM1_HUMAN | 612 | 615 | USERPAT1 | PSAP | X |
| sp\|O14594\|NCAN_HUMAN | 795 | 798 | USERPAT1 | PSAP | |
| sp\|O14513\|NCKP5_HUMAN | 1327 | 1330 | USERPAT1 | PSAP | |
| sp\|Q5T1S8\|NCMAP_HUMAN | 71 | 74 | USERPAT2 | PTAP | |
| sp\|Q9HCD5\|NCOA5_HUMAN | 421 | 424 | USERPAT1 | PSAP | |
| sp\|Q9Y618\|NCOR2_HUMAN | 815 | 818 | USERPAT1 | PSAP | |
| sp\|P52849\|NDST2_HUMAN | 204 | 207 | USERPAT1 | PSAP | |
| sp\|O75438-2\|NDUB1_HUMAN | 7 | 10 | USERPAT1 | PSAP | |
| sp\|Q96PU5\|NED4L_HUMAN | 356 | 359 | USERPAT1 | PSAP | X |
| sp\|Q9H3P2\|NELFA_HUMAN | 226 | 229 | USERPAT2 | PTAP | |
| sp\|Q92859\|NEO1_HUMAN | 439 | 442 | USERPAT1 | PSAP | X |
| sp\|Q9Y4A8\|NF2L3_HUMAN | 504 | 507 | USERPAT2 | PTAP | |
| sp\|O94856\|NFASC_HUMAN | 932 | 935 | USERPAT1 | PSAP | |
| sp\|Q00653\|NFKB2_HUMAN | 423 | 426 | USERPAT1 | PSAP | X |
| sp\|Q6VVB1\|NHLC1_HUMAN | 108 | 111 | USERPAT1 | PSAP | |
| sp\|Q8N5F7\|NKAP_HUMAN | 84 | 87 | USERPAT1 | PSAP | |
| sp\|P26715\|NKG2A_HUMAN | 65 | 68 | USERPAT1 | PSAP | |
| sp\|P43699\|NKX21_HUMAN | 157 | 160 | USERPAT1 | PSAP | |

| | | | | | |
|---|---|---|---|---|---|
| sp\|O60391\|NMD3B_HUMAN | 915 | 918 | USERPAT2 | PTAP | |
| sp\|Q14978\|NOLC1_HUMAN | 295 | 298 | USERPAT1 | PSAP | X |
| sp\|P61580\|NP10_HUMAN | 21 | 24 | USERPAT2 | PTAP | |
| sp\|P61581\|NP24_HUMAN | 21 | 24 | USERPAT2 | PTAP | |
| sp\|P61583\|NP5_HUMAN | 21 | 24 | USERPAT2 | PTAP | |
| sp\|P61582\|NP7_HUMAN | 21 | 24 | USERPAT2 | PTAP | |
| sp\|Q8IUM7\|NPAS4_HUMAN | 376 | 379 | USERPAT1 | PSAP | |
| sp\|E5RHQ5\|NPB11_HUMAN | 314 | 317 | USERPAT1 | PSAP | |
| sp\|A6NJU9\|NPB13_HUMAN | 352 | 355 | USERPAT1 | PSAP | |
| sp\|Q9UND3\|NPIA1_HUMAN | 318 | 321 | USERPAT1 | PSAP | |
| sp\|E9PIF3\|NPIA2_HUMAN | 337 | 340 | USERPAT1 | PSAP | |
| sp\|F8WFD2\|NPIA3_HUMAN | 318 | 321 | USERPAT1 | PSAP | |
| sp\|E9PKD4\|NPIA5_HUMAN | 318 | 321 | USERPAT1 | PSAP | |
| sp\|E9PJI5\|NPIA7_HUMAN | 337 | 340 | USERPAT1 | PSAP | |
| sp\|P0DM63\|NPIA8_HUMAN | 337 | 340 | USERPAT1 | PSAP | |
| sp\|Q92617\|NPIB3_HUMAN | 356 | 359 | USERPAT1 | PSAP | |
| sp\|C9JG80\|NPIB4_HUMAN | 356 | 359 | USERPAT1 | PSAP | |
| sp\|A8MRT5\|NPIB5_HUMAN | 356 | 359 | USERPAT1 | PSAP | |
| sp\|A6NJ64\|NPIL2_HUMAN | 310 | 313 | USERPAT1 | PSAP | |
| sp\|O95436\|NPT2B_HUMAN | 26 | 29 | USERPAT2 | PTAP | |
| sp\|Q92570\|NR4A3_HUMAN | 161 | 164 | USERPAT1 | PSAP | |
| sp\|Q92823\|NRCAM_HUMAN | 952 | 955 | USERPAT1 | PSAP | |
| sp\|Q02297-9\|NRG1_HUMAN | 144 | 147 | USERPAT2 | PTAP | |
| sp\|Q7Z2Y5\|NRK_HUMAN | 1122 | 1125 | USERPAT1 | PSAP | |
| sp\|Q9P2S2\|NRX2A_HUMAN | 1553 | 1556 | USERPAT1 | PSAP | |
| sp\|P58401\|NRX2B_HUMAN | 507 | 510 | USERPAT1 | PSAP | |
| sp\|P49790\|NU153_HUMAN | 1310 | 1313 | USERPAT1 | PSAP | |
| sp\|P35658\|NU214_HUMAN | 1452 | 1455 | USERPAT1 | PSAP | |
| sp\|Q9Y6R0\|NUMBL_HUMAN | 546 | 549 | USERPAT1 | PSAP | X |
| sp\|P37198\|NUP62_HUMAN | 182 | 185 | USERPAT2 | PTAP | |
| sp\|Q16633\|OBF1_HUMAN | 6 | 9 | USERPAT2 | PTAP | |
| sp\|Q5VST9\|OBSCN_HUMAN | 4627 | 4630 | USERPAT1 | PSAP | X |
| sp\|A8MYP8\|ODF3B_HUMAN | 118 | 121 | USERPAT1 | PSAP | |
| sp\|P10515\|ODP2_HUMAN | 340 | 343 | USERPAT1 | PSAP | |
| sp\|O00330\|ODPX_HUMAN | 241 | 244 | USERPAT2 | PTAP | |
| sp\|Q9UHM6\|OPN4_HUMAN | 47 | 50 | USERPAT2 | PTAP | X |
| sp\|Q6IFH4\|OR6B2_HUMAN | 18 | 21 | USERPAT2 | PTAP | |
| sp\|Q8NGW1\|OR6B3_HUMAN | 18 | 21 | USERPAT2 | PTAP | |
| sp\|Q96SN7\|ORAI2_HUMAN | 11 | 14 | USERPAT1 | PSAP | |
| sp\|Q9BZF2\|OSBL7_HUMAN | 225 | 228 | USERPAT1 | PSAP | |
| sp\|Q8IYS5\|OSCAR_HUMAN | 200 | 203 | USERPAT1 | PSAP | |
| sp\|Q8N2R0-3\|OSR2_HUMAN | 113 | 116 | USERPAT1 | PSAP | |
| sp\|Q6ZRI0\|OTOG_HUMAN | 1758 | 1761 | USERPAT1 | PSAP | |
| sp\|Q9Y5P8\|P2R3B_HUMAN | 59 | 62 | USERPAT2 | PTAP | |
| sp\|O75747\|P3C2G_HUMAN | 66 | 69 | USERPAT2 | PTAP | |
| sp\|P11940\|PABP1_HUMAN | 3 | 6 | USERPAT1 | PSAP | X |
| sp\|Q6VY07-2\|PACS1_HUMAN | 985 | 988 | USERPAT2 | PTAP | X |
| sp\|Q9ULE6\|PALD_HUMAN | 849 | 852 | USERPAT1 | PSAP | |
| sp\|A6NDB9\|PALM3_HUMAN | 650 | 653 | USERPAT1 | PSAP | |
| sp\|Q6UXH9\|PAMR1_HUMAN | 654 | 657 | USERPAT2 | PTAP | X |

| | | | | | |
|---|---|---|---|---|---|
| sp\|Q58A45\|PAN3_HUMAN | 426 | 429 | USERPAT2 | PTAP | X |
| sp\|I0J062\|PANO1_HUMAN | 74 | 77 | USERPAT2 | PTAP | |
| sp\|Q9NVV4\|PAPD1_HUMAN | 533 | 536 | USERPAT1 | PSAP | |
| sp\|Q5XG87\|PAPD7_HUMAN | 58 | 61 | USERPAT2 | PTAP | X |
| sp\|Q8TEW0\|PARD3_HUMAN | 432 | 435 | USERPAT1 | PSAP | X |
| sp\|Q6UWI2\|PARM1_HUMAN | 190 | 193 | USERPAT2 | PTAP | |
| sp\|Q06710\|PAX8_HUMAN | 437 | 440 | USERPAT1 | PSAP | X |
| sp\|P57723\|PCBP4_HUMAN | 339 | 342 | USERPAT2 | PTAP | X |
| sp\|Q9Y5F7\|PCDGL_HUMAN | 791 | 794 | USERPAT1 | PSAP | |
| sp\|Q15113\|PCOC1_HUMAN | 310 | 313 | USERPAT1 | PSAP | |
| sp\|Q8WUM4\|PDC6I_HUMAN | 717 | 720 | USERPAT1 | PSAP | X |
| sp\|P50479\|PDLI4_HUMAN | 89 | 92 | USERPAT1 | PSAP | |
| sp\|Q9NR12\|PDLI7_HUMAN | 213 | 216 | USERPAT2 | PTAP | X |
| sp\|Q8IZL8\|PELP1_HUMAN | 491 | 494 | USERPAT1 | PSAP | X |
| sp\|Q5SV97\|PERM1_HUMAN | 525 | 528 | USERPAT1 | PSAP | |
| sp\|O75381\|PEX14_HUMAN | 233 | 236 | USERPAT1 | PSAP | |
| sp\|P98160\|PGBM_HUMAN | 2720 | 2723 | USERPAT1 | PSAP | |
| sp\|Q15124\|PGM5_HUMAN | 12 | 15 | USERPAT2 | PTAP | X |
| sp\|O75167\|PHAR2_HUMAN | 157 | 160 | USERPAT1 | PSAP | |
| sp\|P43119\|PI2R_HUMAN | 336 | 339 | USERPAT1 | PSAP | |
| sp\|Q8IYJ0\|PIANP_HUMAN | 52 | 55 | USERPAT1 | PSAP | |
| sp\|Q92508\|PIEZ1_HUMAN | 373 | 376 | USERPAT2 | PTAP | X |
| sp\|Q9BRB3\|PIGQ_HUMAN | 115 | 118 | USERPAT2 | PTAP | |
| sp\|Q9NWS0-3\|PIHD1_HUMAN | 142 | 145 | USERPAT1 | PSAP | X |
| sp\|Q6IQ23\|PKHA7_HUMAN | 537 | 540 | USERPAT2 | PTAP | X |
| sp\|O94827\|PKHG5_HUMAN | 951 | 954 | USERPAT1 | PSAP | |
| sp\|Q96KN3\|PKNX2_HUMAN | 57 | 60 | USERPAT1 | PSAP | |
| sp\|Q00722\|PLCB2_HUMAN | 487 | 490 | USERPAT1 | PSAP | |
| sp\|Q4KWH8\|PLCH1_HUMAN | 1619 | 1622 | USERPAT1 | PSAP | |
| sp\|Q00G26\|PLIN5_HUMAN | 250 | 253 | USERPAT2 | PTAP | |
| sp\|P00747\|PLMN_HUMAN | 364 | 367 | USERPAT2 | PTAP | |
| sp\|Q8IY17\|PLPL6_HUMAN | 410 | 413 | USERPAT1 | PSAP | X |
| sp\|Q6ZV29\|PLPL7_HUMAN | 379 | 382 | USERPAT1 | PSAP | X |
| sp\|Q9NRQ2\|PLS4_HUMAN | 6 | 9 | USERPAT2 | PTAP | |
| sp\|O43157\|PLXB1_HUMAN | 695 | 698 | USERPAT2 | PTAP | X |
| sp\|Q6XQN6-2\|PNCB_HUMAN | 538 | 541 | USERPAT2 | PTAP | |
| sp\|Q9ULN7\|PNM8B_HUMAN | 186 | 189 | USERPAT1 | PSAP | |
| sp\|Q03052\|PO3F1_HUMAN | 427 | 430 | USERPAT1 | PSAP | |
| sp\|Q8NA72\|POC5_HUMAN | 403 | 406 | USERPAT1 | PSAP | |
| sp\|P63128\|POK9_HUMAN | 253 | 256 | USERPAT2 | PTAP | |
| sp\|Q86T03\|PP4P1_HUMAN | 40 | 43 | USERPAT1 | PSAP | |
| sp\|Q8N4L2\|PP4P2_HUMAN | 23 | 26 | USERPAT2 | PTAP | |
| sp\|Q9UPN7\|PP6R1_HUMAN | 776 | 779 | USERPAT1 | PSAP | X |
| sp\|O75170\|PP6R2_HUMAN | 725 | 728 | USERPAT2 | PTAP | |
| sp\|Q5H9R7\|PP6R3_HUMAN | 861 | 864 | USERPAT1 | PSAP | |
| sp\|Q6NYC8\|PPR18_HUMAN | 438 | 441 | USERPAT2 | PTAP | X |
| sp\|Q5VV67\|PPRC1_HUMAN | 1365 | 1368 | USERPAT1 | PSAP | |
| sp\|Q9HAZ2\|PRD16_HUMAN | 792 | 795 | USERPAT1 | PSAP | X |
| sp\|Q92954\|PRG4_HUMAN | 484 | 487 | USERPAT2 | PTAP | X |
| sp\|Q86YN6\|PRGC2_HUMAN | 143 | 146 | USERPAT1 | PSAP | |

| | | | | | |
|---|---|---|---|---|---|
| sp\|O43900\|PRIC3_HUMAN | 372 | 375 | USERPAT2 | PTAP | |
| sp\|E7EW31\|PROB1_HUMAN | 18 | 21 | USERPAT2 | PTAP | |
| sp\|Q9BWN1\|PRR14_HUMAN | 218 | 221 | USERPAT2 | PTAP | X |
| sp\|Q8IZ63\|PRR22_HUMAN | 108 | 111 | USERPAT1 | PSAP | |
| sp\|Q9H6K5\|PRR36_HUMAN | 377 | 380 | USERPAT1 | PSAP | X |
| sp\|Q8TB68\|PRR7_HUMAN | 217 | 220 | USERPAT1 | PSAP | |
| sp\|Q96M27\|PRRC1_HUMAN | 68 | 71 | USERPAT1 | PSAP | X |
| sp\|C9JH25\|PRRT4_HUMAN | 142 | 145 | USERPAT2 | PTAP | |
| sp\|Q86XN7\|PRSR1_HUMAN | 343 | 346 | USERPAT2 | PTAP | |
| sp\|Q2VWP7\|PRTG_HUMAN | 419 | 422 | USERPAT1 | PSAP | |
| sp\|Q9Y6C5\|PTC2_HUMAN | 262 | 265 | USERPAT1 | PSAP | |
| sp\|Q13308-6\|PTK7_HUMAN | 11 | 14 | USERPAT1 | PSAP | X |
| sp\|Q9H3S7\|PTN23_HUMAN | 720 | 723 | USERPAT2 | PTAP | |
| sp\|P23468\|PTPRD_HUMAN | 609 | 612 | USERPAT1 | PSAP | X |
| sp\|P10586\|PTPRF_HUMAN | 606 | 609 | USERPAT1 | PSAP | X |
| sp\|Q12913\|PTPRJ_HUMAN | 304 | 307 | USERPAT2 | PTAP | X |
| sp\|Q13332\|PTPRS_HUMAN | 618 | 621 | USERPAT1 | PSAP | X |
| sp\|Q96HA9\|PX11C_HUMAN | 158 | 161 | USERPAT2 | PTAP | |
| sp\|Q9BRP8\|PYM1_HUMAN | 125 | 128 | USERPAT1 | PSAP | |
| sp\|P27708\|PYR1_HUMAN | 1822 | 1825 | USERPAT1 | PSAP | |
| sp\|P61106\|RAB14_HUMAN | 194 | 197 | USERPAT1 | PSAP | X |
| sp\|Q9BUV8-5\|RAB5I_HUMAN | 92 | 95 | USERPAT1 | PSAP | |
| sp\|Q3YEC7\|RABL6_HUMAN | 348 | 351 | USERPAT1 | PSAP | |
| sp\|Q96EV2\|RBM33_HUMAN | 700 | 703 | USERPAT2 | PTAP | |
| sp\|Q5TC82\|RC3H1_HUMAN | 629 | 632 | USERPAT1 | PSAP | X |
| sp\|Q8IZ40\|RCOR2_HUMAN | 458 | 461 | USERPAT2 | PTAP | X |
| sp\|Q9P2K3\|RCOR3_HUMAN | 404 | 407 | USERPAT2 | PTAP | |
| sp\|O75452\|RDH16_HUMAN | 118 | 121 | USERPAT2 | PTAP | |
| sp\|Q01201\|RELB_HUMAN | 493 | 496 | USERPAT2 | PTAP | |
| sp\|Q8IYK8\|REM2_HUMAN | 57 | 60 | USERPAT1 | PSAP | X |
| sp\|Q9P2R6\|RERE_HUMAN | 791 | 794 | USERPAT2 | PTAP | |
| sp\|O75154\|RFIP3_HUMAN | 87 | 90 | USERPAT1 | PSAP | |
| sp\|Q96B86\|RGMA_HUMAN | 349 | 352 | USERPAT2 | PTAP | |
| sp\|Q6ZVN8\|RGMC_HUMAN | 124 | 127 | USERPAT2 | PTAP | |
| sp\|Q96CC6\|RHDF1_HUMAN | 532 | 535 | USERPAT1 | PSAP | X |
| sp\|Q9BRR9\|RHG09_HUMAN | 365 | 368 | USERPAT2 | PTAP | X |
| sp\|Q68EM7\|RHG17_HUMAN | 689 | 692 | USERPAT1 | PSAP | X |
| sp\|Q5T5U3\|RHG21_HUMAN | 207 | 210 | USERPAT1 | PSAP | X |
| sp\|A7KAX9\|RHG32_HUMAN | 1956 | 1959 | USERPAT1 | PSAP | |
| sp\|Q86UR5\|RIMS1_HUMAN | 235 | 238 | USERPAT1 | PSAP | |
| sp\|Q8TB24\|RIN3_HUMAN | 383 | 386 | USERPAT2 | PTAP | |
| sp\|P26373\|RL13_HUMAN | 131 | 134 | USERPAT1 | PSAP | |
| sp\|O94763\|RMP_HUMAN | 15 | 18 | USERPAT1 | PSAP | |
| sp\|Q9NRR4\|RNC_HUMAN | 29 | 32 | USERPAT1 | PSAP | |
| sp\|Q9H0F5\|RNF38_HUMAN | 337 | 340 | USERPAT1 | PSAP | |
| sp\|Q7L0R7\|RNF44_HUMAN | 13 | 16 | USERPAT1 | PSAP | |
| sp\|Q9Y6N7\|ROBO1_HUMAN | 561 | 564 | USERPAT1 | PSAP | |
| sp\|Q9HCK4\|ROBO2_HUMAN | 736 | 739 | USERPAT1 | PSAP | X |
| sp\|Q96MS0\|ROBO3_HUMAN | 856 | 859 | USERPAT1 | PSAP | |
| sp\|Q8WZ75\|ROBO4_HUMAN | 347 | 350 | USERPAT1 | PSAP | |

| | | | | | |
|---|---|---|---|---|---|
| sp\|P08922\|ROS1_HUMAN | 102 | 105 | USERPAT2 | PTAP | |
| sp\|P10301\|RRAS_HUMAN | 203 | 206 | USERPAT1 | PSAP | X |
| sp\|O95197\|RTN3_HUMAN | 22 | 25 | USERPAT1 | PSAP | X |
| sp\|Q9NQC3\|RTN4_HUMAN | 69 | 72 | USERPAT2 | PTAP | X |
| sp\|Q9H714-4\|RUBCL_HUMAN | 588 | 591 | USERPAT1 | PSAP | |
| sp\|Q8N1F8\|S11IP_HUMAN | 453 | 456 | USERPAT1 | PSAP | |
| sp\|P55011\|S12A2_HUMAN | 5 | 8 | USERPAT2 | PTAP | |
| sp\|Q92503\|S14L1_HUMAN | 225 | 228 | USERPAT1 | PSAP | |
| sp\|Q8N808\|S35G3_HUMAN | 19 | 22 | USERPAT1 | PSAP | |
| sp\|Q96KT7\|S35G5_HUMAN | 19 | 22 | USERPAT1 | PSAP | |
| sp\|P0C7Q6\|S35G6_HUMAN | 19 | 22 | USERPAT1 | PSAP | |
| sp\|Q9NSD5\|S6A13_HUMAN | 582 | 585 | USERPAT1 | PSAP | |
| sp\|Q9BXA9\|SALL3_HUMAN | 248 | 251 | USERPAT1 | PSAP | |
| sp\|P0C264\|SBK3_HUMAN | 202 | 205 | USERPAT2 | PTAP | |
| sp\|O15027\|SC16A_HUMAN | 1773 | 1776 | USERPAT1 | PSAP | X |
| sp\|O94979\|SC31A_HUMAN | 1040 | 1043 | USERPAT1 | PSAP | X |
| sp\|O14828\|SCAM3_HUMAN | 64 | 67 | USERPAT1 | PSAP | X |
| sp\|Q8N228\|SCML4_HUMAN | 300 | 303 | USERPAT1 | PSAP | |
| sp\|P51172\|SCNND_HUMAN | 34 | 37 | USERPAT1 | PSAP | |
| sp\|Q7Z5N4\|SDK1_HUMAN | 768 | 771 | USERPAT1 | PSAP | X |
| sp\|Q58EX2\|SDK2_HUMAN | 690 | 693 | USERPAT2 | PTAP | X |
| sp\|Q6UXD5\|SE6L2_HUMAN | 117 | 120 | USERPAT2 | PTAP | X |
| sp\|Q9BVL4\|SELO_HUMAN | 71 | 74 | USERPAT1 | PSAP | |
| sp\|Q13275\|SEM3F_HUMAN | 178 | 181 | USERPAT2 | PTAP | X |
| sp\|Q9H3S1\|SEM4A_HUMAN | 315 | 318 | USERPAT2 | PTAP | |
| sp\|O75326\|SEM7A_HUMAN | 11 | 14 | USERPAT1 | PSAP | |
| sp\|Q9UHD8\|SEPT9_HUMAN | 176 | 179 | USERPAT2 | PTAP | |
| sp\|P84101-2\|SERF2_HUMAN | 75 | 78 | USERPAT1 | PSAP | X |
| sp\|Q8N4B1\|SESQ1_HUMAN | 163 | 166 | USERPAT1 | PSAP | |
| sp\|O15047\|SET1A_HUMAN | 1127 | 1130 | USERPAT1 | PSAP | X |
| sp\|Q9UPS6\|SET1B_HUMAN | 647 | 650 | USERPAT1 | PSAP | X |
| sp\|Q15047\|SETB1_HUMAN | 453 | 456 | USERPAT2 | PTAP | |
| sp\|Q9C0A6\|SETD5_HUMAN | 1054 | 1057 | USERPAT1 | PSAP | X |
| sp\|Q15459\|SF3A1_HUMAN | 633 | 636 | USERPAT1 | PSAP | X |
| sp\|Q15428\|SF3A2_HUMAN | 416 | 419 | USERPAT1 | PSAP | X |
| sp\|Q15427\|SF3B4_HUMAN | 218 | 221 | USERPAT1 | PSAP | X |
| sp\|Q5HYK7\|SH319_HUMAN | 157 | 160 | USERPAT1 | PSAP | |
| sp\|Q96B97\|SH3K1_HUMAN | 343 | 346 | USERPAT1 | PSAP | X |
| sp\|Q9Y566\|SHAN1_HUMAN | 474 | 477 | USERPAT1 | PSAP | X |
| sp\|P29353\|SHC1_HUMAN | 379 | 382 | USERPAT2 | PTAP | X |
| sp\|Q92529\|SHC3_HUMAN | 113 | 116 | USERPAT1 | PSAP | |
| sp\|O15357\|SHIP2_HUMAN | 166 | 169 | USERPAT2 | PTAP | X |
| sp\|Q8TBC3\|SHKB1_HUMAN | 612 | 615 | USERPAT1 | PSAP | |
| sp\|Q9UHJ6\|SHPK_HUMAN | 300 | 303 | USERPAT2 | PTAP | X |
| sp\|P78324\|SHPS1_HUMAN | 148 | 151 | USERPAT1 | PSAP | X |
| sp\|Q6IA17\|SIGIR_HUMAN | 366 | 369 | USERPAT1 | PSAP | X |
| sp\|O00241\|SIRB1_HUMAN | 147 | 150 | USERPAT1 | PSAP | X |
| sp\|Q5TFQ8\|SIRBL_HUMAN | 147 | 150 | USERPAT1 | PSAP | |
| sp\|Q9P1W8\|SIRPG_HUMAN | 146 | 149 | USERPAT1 | PSAP | |
| sp\|P84550\|SKOR1_HUMAN | 680 | 683 | USERPAT1 | PSAP | |

| | | | | | |
|---|---|---|---|---|---|
| sp\|Q9P270\|SLAI2_HUMAN | 539 | 542 | USERPAT1 | PSAP | |
| sp\|Q8IW52\|SLIK4_HUMAN | 587 | 590 | USERPAT1 | PSAP | |
| sp\|Q9NZC9\|SMAL1_HUMAN | 323 | 326 | USERPAT1 | PSAP | |
| sp\|Q96Q15\|SMG1_HUMAN | 1815 | 1818 | USERPAT2 | PTAP | X |
| sp\|Q9H0W8\|SMG9_HUMAN | 82 | 85 | USERPAT2 | PTAP | |
| sp\|Q99835\|SMO_HUMAN | 750 | 753 | USERPAT1 | PSAP | X |
| sp\|Q8TAQ2\|SMRC2_HUMAN | 1199 | 1202 | USERPAT2 | PTAP | X |
| sp\|Q6STE5\|SMRD3_HUMAN | 89 | 92 | USERPAT2 | PTAP | X |
| sp\|Q53GS9\|SNUT2_HUMAN | 396 | 399 | USERPAT2 | PTAP | X |
| sp\|Q96L92\|SNX27_HUMAN | 11 | 14 | USERPAT1 | PSAP | X |
| sp\|Q6ZQN7\|SO4C1_HUMAN | 64 | 67 | USERPAT1 | PSAP | X |
| sp\|Q9H2Y9\|SO5A1_HUMAN | 83 | 86 | USERPAT1 | PSAP | |
| sp\|Q8IXZ3\|SP8_HUMAN | 311 | 314 | USERPAT1 | PSAP | |
| sp\|Q96R06\|SPAG5_HUMAN | 390 | 393 | USERPAT1 | PSAP | X |
| sp\|Q495Y8\|SPDE2_HUMAN | 52 | 55 | USERPAT1 | PSAP | |
| sp\|A6NKU9\|SPDE3_HUMAN | 34 | 37 | USERPAT1 | PSAP | |
| sp\|A6NLX3\|SPDE4_HUMAN | 35 | 38 | USERPAT1 | PSAP | |
| sp\|A6NIY4\|SPDE5_HUMAN | 52 | 55 | USERPAT1 | PSAP | |
| sp\|P0CI01\|SPDE6_HUMAN | 52 | 55 | USERPAT1 | PSAP | |
| sp\|A6NHP3\|SPE2B_HUMAN | 52 | 55 | USERPAT1 | PSAP | |
| sp\|Q15772\|SPEG_HUMAN | 2181 | 2184 | USERPAT1 | PSAP | |
| sp\|Q9NYA1-2\|SPHK1_HUMAN | 71 | 74 | USERPAT2 | PTAP | |
| sp\|O43291\|SPIT2_HUMAN | 111 | 114 | USERPAT1 | PSAP | X |
| sp\|Q96N96-4\|SPT13_HUMAN | 550 | 553 | USERPAT2 | PTAP | |
| sp\|Q6ZRS2\|SRCAP_HUMAN | 1560 | 1563 | USERPAT1 | PSAP | |
| sp\|Q9C0H9\|SRCN1_HUMAN | 610 | 613 | USERPAT1 | PSAP | |
| sp\|A1L4H1\|SRCRL_HUMAN | 413 | 416 | USERPAT2 | PTAP | |
| sp\|Q9UQ35\|SRRM2_HUMAN | 2290 | 2293 | USERPAT2 | PTAP | X |
| sp\|Q14140\|SRTD2_HUMAN | 151 | 154 | USERPAT2 | PTAP | |
| sp\|Q9UGK3\|STAP2_HUMAN | 271 | 274 | USERPAT1 | PSAP | |
| sp\|Q9P2P6\|STAR9_HUMAN | 3060 | 3063 | USERPAT1 | PSAP | X |
| sp\|O76061\|STC2_HUMAN | 219 | 222 | USERPAT2 | PTAP | X |
| sp\|Q13586\|STIM1_HUMAN | 601 | 604 | USERPAT1 | PSAP | X |
| sp\|Q9NRP7\|STK36_HUMAN | 375 | 378 | USERPAT1 | PSAP | |
| sp\|Q8N7X2-4\|STPG3_HUMAN | 284 | 287 | USERPAT1 | PSAP | |
| sp\|Q9Y3F4\|STRAP_HUMAN | 343 | 346 | USERPAT1 | PSAP | X |
| sp\|Q13033\|STRN3_HUMAN | 162 | 165 | USERPAT2 | PTAP | X |
| sp\|Q8TCJ2\|STT3B_HUMAN | 4 | 7 | USERPAT1 | PSAP | X |
| sp\|Q9UMX1\|SUFU_HUMAN | 12 | 15 | USERPAT2 | PTAP | |
| sp\|Q8IWZ8\|SUGP1_HUMAN | 110 | 113 | USERPAT1 | PSAP | |
| sp\|Q4LDE5\|SVEP1_HUMAN | 2720 | 2723 | USERPAT2 | PTAP | X |
| sp\|Q7L8C5\|SYT13_HUMAN | 108 | 111 | USERPAT2 | PTAP | |
| sp\|Q8NBV8\|SYT8_HUMAN | 22 | 25 | USERPAT1 | PSAP | |
| sp\|O76070\|SYUG_HUMAN | 102 | 105 | USERPAT1 | PSAP | |
| sp\|Q9BXQ6\|T121B_HUMAN | 538 | 541 | USERPAT1 | PSAP | |
| sp\|Q6IEE7\|T132E_HUMAN | 967 | 970 | USERPAT2 | PTAP | |
| sp\|Q7Z7N9\|T179B_HUMAN | 57 | 60 | USERPAT1 | PSAP | |
| sp\|Q15714\|T22D1_HUMAN | 845 | 848 | USERPAT1 | PSAP | |
| sp\|O95359\|TACC2_HUMAN | 1921 | 1924 | USERPAT1 | PSAP | X |
| sp\|Q15572\|TAF1C_HUMAN | 576 | 579 | USERPAT1 | PSAP | |

| | | | | | |
|---|---|---|---|---|---|
| sp\|Q9Y6J9\|TAF6L_HUMAN | 463 | 466 | USERPAT2 | PTAP | X |
| sp\|Q86VP1\|TAXB1_HUMAN | 701 | 704 | USERPAT2 | PTAP | X |
| sp\|Q9C0C2\|TB182_HUMAN | 336 | 339 | USERPAT1 | PSAP | |
| sp\|Q9ULP9\|TBC24_HUMAN | 463 | 466 | USERPAT2 | PTAP | X |
| sp\|Q9Y2I9\|TBC30_HUMAN | 849 | 852 | USERPAT1 | PSAP | X |
| sp\|Q9UL17\|TBX21_HUMAN | 410 | 413 | USERPAT1 | PSAP | |
| sp\|O95947\|TBX6_HUMAN | 79 | 82 | USERPAT1 | PSAP | |
| sp\|O95947-2\|TBX6_HUMAN | 79 | 82 | USERPAT1 | PSAP | |
| sp\|Q9Y242\|TCF19_HUMAN | 215 | 218 | USERPAT1 | PSAP | |
| sp\|Q7Z6L1\|TCPR1_HUMAN | 1153 | 1156 | USERPAT1 | PSAP | |
| sp\|Q5VWI1\|TCRGL_HUMAN | 94 | 97 | USERPAT1 | PSAP | |
| sp\|Q9H7E2\|TDRD3_HUMAN | 267 | 270 | USERPAT1 | PSAP | |
| sp\|Q9Y2W6\|TDRKH_HUMAN | 246 | 249 | USERPAT2 | PTAP | |
| sp\|Q15561\|TEAD4_HUMAN | 206 | 209 | USERPAT1 | PSAP | |
| sp\|Q6NUS6\|TECT3_HUMAN | 73 | 76 | USERPAT1 | PSAP | |
| sp\|Q15569\|TESK1_HUMAN | 556 | 559 | USERPAT1 | PSAP | X |
| sp\|Q8NFU7\|TET1_HUMAN | 1835 | 1838 | USERPAT1 | PSAP | |
| sp\|O43151\|TET3_HUMAN | 419 | 422 | USERPAT1 | PSAP | |
| sp\|Q8IWB9\|TEX2_HUMAN | 18 | 21 | USERPAT1 | PSAP | |
| sp\|Q53QW1\|TEX44_HUMAN | 149 | 152 | USERPAT1 | PSAP | |
| sp\|A6NCN8\|TEX52_HUMAN | 258 | 261 | USERPAT1 | PSAP | |
| sp\|Q8WUA4\|TF3C2_HUMAN | 120 | 123 | USERPAT1 | PSAP | |
| sp\|Q92734\|TFG_HUMAN | 196 | 199 | USERPAT1 | PSAP | X |
| sp\|Q9Y4F4\|TGRM1_HUMAN | 28 | 31 | USERPAT1 | PSAP | |
| sp\|Q8WY91\|THAP4_HUMAN | 300 | 303 | USERPAT1 | PSAP | X |
| sp\|Q5TEJ8\|THMS2_HUMAN | 172 | 175 | USERPAT1 | PSAP | X |
| sp\|Q9NS62\|THSD1_HUMAN | 568 | 571 | USERPAT1 | PSAP | X |
| sp\|O95411\|TIAF1_HUMAN | 56 | 59 | USERPAT1 | PSAP | |
| sp\|P49788\|TIG1_HUMAN | 20 | 23 | USERPAT2 | PTAP | X |
| sp\|Q495A1\|TIGIT_HUMAN | 191 | 194 | USERPAT1 | PSAP | |
| sp\|A6NFA1\|TIKI2_HUMAN | 394 | 397 | USERPAT2 | PTAP | |
| sp\|Q8WZ42\|TITIN_HUMAN | 14415 | 14418 | USERPAT1 | PSAP | |
| sp\|O75674\|TM1L1_HUMAN | 179 | 182 | USERPAT2 | PTAP | X |
| sp\|C9JI98\|TM238_HUMAN | 18 | 21 | USERPAT1 | PSAP | |
| sp\|Q9BX74\|TM2D1_HUMAN | 9 | 12 | USERPAT1 | PSAP | X |
| sp\|Q9BSE2\|TMM79_HUMAN | 84 | 87 | USERPAT1 | PSAP | |
| sp\|A0PJX8\|TMM82_HUMAN | 332 | 335 | USERPAT1 | PSAP | |
| sp\|P21580\|TNAP3_HUMAN | 462 | 465 | USERPAT2 | PTAP | |
| sp\|Q15025\|TNIP1_HUMAN | 100 | 103 | USERPAT2 | PTAP | X |
| sp\|Q8NDV7\|TNR6A_HUMAN | 240 | 243 | USERPAT1 | PSAP | X |
| sp\|Q9UPQ9\|TNR6B_HUMAN | 1739 | 1742 | USERPAT1 | PSAP | |
| sp\|Q96NM4-4\|TOX2_HUMAN | 7 | 10 | USERPAT1 | PSAP | |
| sp\|P48553\|TPC10_HUMAN | 886 | 889 | USERPAT1 | PSAP | |
| sp\|Q6ZTW0\|TPGS1_HUMAN | 265 | 268 | USERPAT1 | PSAP | |
| sp\|P40225\|TPO_HUMAN | 327 | 330 | USERPAT1 | PSAP | |
| sp\|Q9Y4K3\|TRAF6_HUMAN | 236 | 239 | USERPAT2 | PTAP | X |
| sp\|Q7Z4K8-3\|TRI46_HUMAN | 476 | 479 | USERPAT1 | PSAP | |
| sp\|Q96LD4\|TRI47_HUMAN | 99 | 102 | USERPAT1 | PSAP | X |
| sp\|Q96RU7\|TRIB3_HUMAN | 53 | 56 | USERPAT2 | PTAP | |
| sp\|Q9C037\|TRIM4_HUMAN | 55 | 58 | USERPAT1 | PSAP | |

| | | | | | |
|---|---|---|---|---|---|
| sp\|O75962\|TRIO_HUMAN | 2387 | 2390 | USERPAT1 | PSAP | X |
| sp\|Q9HCF6\|TRPM3_HUMAN | 1668 | 1671 | USERPAT1 | PSAP | |
| sp\|Q9Y4A5\|TRRAP_HUMAN | 488 | 491 | USERPAT2 | PTAP | X |
| sp\|P07477\|TRY1_HUMAN | 129 | 132 | USERPAT2 | PTAP | |
| sp\|P07478\|TRY2_HUMAN | 129 | 132 | USERPAT2 | PTAP | |
| sp\|Q8NHM4\|TRY6_HUMAN | 129 | 132 | USERPAT2 | PTAP | |
| sp\|Q99816\|TS101_HUMAN | 320 | 323 | USERPAT2 | PTAP | X |
| sp\|P50607\|TUB_HUMAN | 101 | 104 | USERPAT2 | PTAP | |
| sp\|Q9Y6I9\|TX264_HUMAN | 305 | 308 | USERPAT2 | PTAP | |
| sp\|Q8NBS9\|TXND5_HUMAN | 182 | 185 | USERPAT1 | PSAP | X |
| sp\|Q9NPG3\|UBN1_HUMAN | 723 | 726 | USERPAT1 | PSAP | |
| sp\|O95071\|UBR5_HUMAN | 1493 | 1496 | USERPAT2 | PTAP | |
| sp\|Q9BZM4\|ULBP3_HUMAN | 208 | 211 | USERPAT2 | PTAP | |
| sp\|Q8IZJ1\|UNC5B_HUMAN | 40 | 43 | USERPAT1 | PSAP | |
| sp\|Q6UXZ4\|UNC5D_HUMAN | 47 | 50 | USERPAT1 | PSAP | X |
| sp\|O75445\|USH2A_HUMAN | 4529 | 4532 | USERPAT1 | PSAP | |
| sp\|P46939\|UTRO_HUMAN | 869 | 872 | USERPAT1 | PSAP | X |
| sp\|P50552\|VASP_HUMAN | 248 | 251 | USERPAT2 | PTAP | X |
| sp\|P52735\|VAV2_HUMAN | 805 | 808 | USERPAT1 | PSAP | |
| sp\|O43915\|VEGFD_HUMAN | 193 | 196 | USERPAT2 | PTAP | |
| sp\|Q69YN4\|VIR_HUMAN | 79 | 82 | USERPAT1 | PSAP | |
| sp\|Q5THJ4\|VP13D_HUMAN | 3069 | 3072 | USERPAT1 | PSAP | X |
| sp\|Q9H9H4\|VP37B_HUMAN | 185 | 188 | USERPAT2 | PTAP | X |
| sp\|Q9UK41-2\|VPS28_HUMAN | 211 | 214 | USERPAT1 | PSAP | X |
| sp\|Q9UID3\|VPS51_HUMAN | 666 | 669 | USERPAT1 | PSAP | X |
| sp\|Q8N0Z9\|VSI10_HUMAN | 225 | 228 | USERPAT1 | PSAP | |
| sp\|P0DP72\|VSXL2_HUMAN | 371 | 374 | USERPAT2 | PTAP | |
| sp\|Q96DN2\|VWCE_HUMAN | 330 | 333 | USERPAT1 | PSAP | |
| sp\|A8K0Z3\|WASH1_HUMAN | 272 | 275 | USERPAT1 | PSAP | |
| sp\|Q6VEQ5\|WASH2_HUMAN | 272 | 275 | USERPAT1 | PSAP | |
| sp\|C4AMC7\|WASH3_HUMAN | 270 | 273 | USERPAT1 | PSAP | |
| sp\|A8MWX3\|WASH4_HUMAN | 285 | 288 | USERPAT1 | PSAP | |
| sp\|Q9NQA3\|WASH6_HUMAN | 254 | 257 | USERPAT1 | PSAP | |
| sp\|Q8IZU2\|WDR17_HUMAN | 278 | 281 | USERPAT1 | PSAP | |
| sp\|Q9H7D7\|WDR26_HUMAN | 61 | 64 | USERPAT1 | PSAP | X |
| sp\|A2RRH5\|WDR27_HUMAN | 807 | 810 | USERPAT1 | PSAP | |
| sp\|Q9Y4E6\|WDR7_HUMAN | 969 | 972 | USERPAT1 | PSAP | X |
| sp\|Q6ZMY6\|WDR88_HUMAN | 21 | 24 | USERPAT1 | PSAP | |
| sp\|A6NGB9\|WIPF3_HUMAN | 26 | 29 | USERPAT1 | PSAP | |
| sp\|P04628\|WNT1_HUMAN | 105 | 108 | USERPAT2 | PTAP | |
| sp\|Q5GH76\|XKR4_HUMAN | 577 | 580 | USERPAT2 | PTAP | |
| sp\|Q6P2D8\|XRRA1_HUMAN | 593 | 596 | USERPAT2 | PTAP | |
| sp\|Q9Y2T7\|YBOX2_HUMAN | 58 | 61 | USERPAT1 | PSAP | X |
| sp\|P49750\|YLPM1_HUMAN | 250 | 253 | USERPAT1 | PSAP | |
| sp\|Q6UXP9\|YO001_HUMAN | 81 | 84 | USERPAT2 | PTAP | |
| sp\|Q6ZRN7\|YP029_HUMAN | 180 | 183 | USERPAT1 | PSAP | |
| sp\|A8MZ25\|YQ037_HUMAN | 50 | 53 | USERPAT2 | PTAP | |
| sp\|A8MWP4\|YU008_HUMAN | 22 | 25 | USERPAT1 | PSAP | |
| sp\|Q8ND82\|Z280C_HUMAN | 524 | 527 | USERPAT2 | PTAP | |
| sp\|Q13105\|ZBT17_HUMAN | 793 | 796 | USERPAT2 | PTAP | |

| | | | | | |
|---|---|---|---|---|---|
| sp\|Q96K62\|ZBT45_HUMAN | 346 | 349 | USERPAT1 | PSAP | |
| sp\|Q8WYQ9\|ZCH14_HUMAN | 365 | 368 | USERPAT1 | PSAP | X |
| sp\|Q9C0B5\|ZDHC5_HUMAN | 626 | 629 | USERPAT2 | PTAP | X |
| sp\|Q5T1R4\|ZEP3_HUMAN | 1023 | 1026 | USERPAT1 | PSAP | X |
| sp\|Q9C0A1\|ZFHX2_HUMAN | 1226 | 1229 | USERPAT1 | PSAP | |
| sp\|Q86UP3\|ZFHX4_HUMAN | 2016 | 2019 | USERPAT1 | PSAP | |
| sp\|Q68DK2\|ZFY26_HUMAN | 956 | 959 | USERPAT2 | PTAP | X |
| sp\|P0C6A0\|ZGLP1_HUMAN | 119 | 122 | USERPAT1 | PSAP | |
| sp\|Q96T25\|ZIC5_HUMAN | 147 | 150 | USERPAT1 | PSAP | |
| sp\|Q15776\|ZKSC8_HUMAN | 8 | 11 | USERPAT1 | PSAP | |
| sp\|Q9H091\|ZMY15_HUMAN | 718 | 721 | USERPAT1 | PSAP | |
| sp\|Q14202\|ZMYM3_HUMAN | 813 | 816 | USERPAT2 | PTAP | |
| sp\|Q5VUA4\|ZN318_HUMAN | 743 | 746 | USERPAT1 | PSAP | X |
| sp\|Q96JG9\|ZN469_HUMAN | 3810 | 3813 | USERPAT1 | PSAP | |
| sp\|Q96F45\|ZN503_HUMAN | 323 | 326 | USERPAT1 | PSAP | X |
| sp\|Q86XF7\|ZN575_HUMAN | 202 | 205 | USERPAT2 | PTAP | |
| sp\|Q5EBL2\|ZN628_HUMAN | 444 | 447 | USERPAT1 | PSAP | |
| sp\|Q8N1G0\|ZN687_HUMAN | 179 | 182 | USERPAT1 | PSAP | |
| sp\|Q96CS4\|ZN689_HUMAN | 4 | 7 | USERPAT1 | PSAP | |
| sp\|Q6XR72\|ZNT10_HUMAN | 182 | 185 | USERPAT2 | PTAP | |
| sp\|A7E2V4\|ZSWM8_HUMAN | 1047 | 1050 | USERPAT1 | PSAP | |
